# National practice of Nursing professionals in the insertion of peripheral vascular access devices [Fn fn01b]

**DOI:** 10.1590/1518-8345.6673.4314

**Published:** 2024-09-23

**Authors:** Bianka Sousa Martins Silva, Luciano Marques dos Santos, Patrícia Kuerten Rocha, Aline Nair Biaggio Mota, Ariane Ferreira Machado Avelar, Denise Miyuki Kusahara

**Affiliations:** 1 Universidade Federal de São Paulo, Escola Paulista de Enfermagem, São Paulo, SP, Brazil; 2 Universidade Estadual de Feira de Santana, Departamento de Saúde, Feira de Santana, BA, Brazil.; 3 Universidade Federal de Santa Catarina, Departamento de Saúde, Santa Catarina, SC, Brazil.; 4 BIOTRONIK Comercial Médica, Medical Affairs, São Paulo, SP, Brazil.

**Keywords:** Peripheral Catheterization, Intravenous Infusions, Vascular Access Devices, Nursing Team, Nursing Care, Professional Competence

## Abstract

**Objective:**

to identify and compare the practice of Nursing professionals regarding the insertion of peripheral vascular access devices, according to professional category.

**Method:**

descriptive sectional study carried out between July 2021 and May 2022 with 2,584 Nursing professionals, using a questionnaire validated by three judges with expertise in intravenous therapy, containing variables related to catheterization and the vascular access device. Descriptive and inferential analysis was carried out.

**Results:**

most professionals do not prepare the patient or perform some essential care before attempting peripheral intravenous catheterization. Regarding the preferred catheterization site, hands, arm and forearm stand out. There is no control over the tourniquet time, and the patient is punctured more than three times. The most used device materials are polyurethane and Teflon ^®^ , more than one criterion is adopted for device selection, and Micropore ^®^ type adhesive tape was the covering most cited by Nursing professionals. The identification of catheterization was not adequate.

**Conclusion:**

Nursing technicians and assistants are the professionals who least comply with what is recommended in recognized guidelines. Nurses’ practice also presents deviations from scientific evidence.

## Highlights

**(1)** Most Nursing professionals do not involve the patient and the family in care.

**(2)** Technicians/assistants are those who least meet the recommended standards.

**(3)** Nurses also present deviations in relation to scientific evidence.

**(4)** The nurse’s performance was not very different from professionals with a secondary level.

**(5)** There are weaknesses in the care offered to the patient.

## Introduction

 Peripheral intravenous catheterization (PIC) is extensively performed on patients in a wide range of healthcare settings, mainly for the insertion of vascular access devices (VAD). This procedure enables the implementation of various therapies, such as the administration of fluids, medications, blood components and nutrition directly into the circulatory system ^(^ 1 ^)^ . 

 Multinational cross-sectional research carried out in five Latin American countries indicates that around 70% of hospitalized patients undergo PIC ^(^ 2 ^)^ . Although routine, this practice can cause complications that result in morbidity and mortality, increased hospitalization time and significant costs that impact the patient’s clinical status and evolution ^(^ 3 ^)^ . VAD (re)insertion is a stressful procedure for the patient and family during hospitalization, and is associated with increased needle phobia and resistance among adults in seeking care in health services ^(^ 4 ^-^ 5 ^)^ . 

 The complexity of PIC and its high rate of associated complications indicate that the nurse should be the protagonist and act in all stages of the process, from selection of the type, installation, to removal of the VAD, in line with recognized international recommendations and guidelines for Nursing practice ^(^ 6 ^-^ 7 ^)^ . 

 In the workplace, all peripheral VAD insertion Nursing professionals must be trained effectively to provide patients with high-quality care guided by the best evidence ^(^ 8 ^)^ . 

 Knowing the peripheral VAD insertion practices performed by Nursing professionals is imperative to guarantee the effectiveness of the treatment and care provided, in addition to avoiding the emergence of complications related to intravenous therapy (IVT). Evidence indicates that in the period from June 2021 to May 2022, around 39,994 adverse events/complications involving vascular access devices were reported in health services ^(^ 9 ^)^ . 

Thus, considering the importance of PIC and the need to evaluate the execution of this procedure, with the aim of improving the quality of care, the objective of this study is to identify and compare the practice of Nursing professionals regarding the insertion of peripheral vascular access devices, according to professional category.

## Method

### Study design

This is a sectional study of the descriptive survey type. To describe and report the study, the Strengthening the Reporting of Observational Studies in Epidemiology (STROBE) was used as a reference.

### Location

Held in the five macro-regions of Brazil.

### Period

Data collection was carried out between July 2021 and May 2022.

### Population and selection criteria

 The study population were nurses, technicians and nursing assistants residing in the five macro-regions of Brazil. Data from the *Conselho Federal de Enfermagem* (Cofen) (2022) registered a total of 2,513,428 Nursing professionals, of which 613,827 (24.42%) were nurses, 1,463,072 (58.21%) nursing technicians and 436,529 (17. 37%) nursing assistants ^(^ 10 ^)^ . 

Professionals who worked in direct care for patients undergoing PIC and who performed this procedure during their routine activities were included. Professionals with up to one year after completing their undergraduate or technical course and without prior experience in PIC were not included, and those who did not inform their professional category in the questionnaire were excluded.

### Sample

 For the sample calculation, a population of 2,513,428 Nursing professionals was considered, with a precision of 3% and a frequency of 80% of PIC. The minimum sample was estimated at 837 participants. The sample size calculation was carried out using the OpenEpi tool, available free of charge on the internet ( https://www.openepi.com/Menu/OE_Menu.htm ). 

 Initially, an e-mail message was sent to Cofen requesting access to information from pediatric nurses in Brazil. The request generated the Administrative Process COFEN nº 0940/2017, which was forwarded to the General Procuracy Sector. This issued legal opinion nº 10/2018, in favor of providing the data after formalizing a Commitment Term between Cofen and the *Universidade Federal de São Paulo* / *Programa de Pós-Graduação em Enfermagem* . 

Cofen has made available a database containing 613,987 active registrations of nurses and nursing technicians. The only information present was the “professional category” and “electronic address (e-mail)”, which made it difficult to identify pediatric nurses. For this reason, the population was expanded to include children and adults.

 Furthermore, there was also a lot of duplicate data and inconsistencies, such as the same email being repeated around 16,644 times (2.71%). In this way, an intentional non-probabilistic sample was used, which included a chain-referral or snowball phase ^(^ 11 ^)^ . This technique was used in a complementary way because the Cofen data did not represent the Nursing population. 

 The first step in the snowball sampling method was to find individuals belonging to the study’s target population through social networks (Instagram ^®^ , Facebook ^®^ ) and WhatsApp ^®^ , research groups and the researchers’ contact professionals. 

These professionals were called the seed of the sample, and gave rise to other sampled professionals. From the seed, the snowball process began, in which the first professionals were considered wave zero. The professionals indicated by wave zero who were part of the target population and who were not part of wave zero constituted wave one. These individuals were asked to forward the research link to other professionals, and so on.

### Study variables

Variables related to the professional were investigated (professional category, education time and number of peripheral VAD inserted in a 12-hour work shift), as well as variables related to PIC (explains the procedure to the family member/guardian and patient, strategy and preparation of the patient, resource for pain management before catheterization), variables related to PIC attempts (sanitizes hands, changes gloves after each attempt, uses new material for antisepsis with each catheterization attempt, changes the device with each PIC attempt), variables related to VAD insertion (site of venous catheterization in children and adults, device insertion method, method for evaluating the venous network, use of a clinical tool for evaluating difficult venous networks, criteria for selecting peripheral veins, number of attempts of catheterization, limb tourniquet, tourniquet time and distance from the tourniquet to the catheterization area), variables related to the device and covering (type and material of the device, caliber of the device used in children and adults, criteria for selecting the device and material used for device covering/stabilization) and variables related to registration (PIC identification).

### Data collection and instruments

 Data collection was carried out using a questionnaire containing questions about patient preparation, care performed by Nursing professionals before PIC, method of evaluation and selection of peripheral veins, technique and procedures adopted for insertion of the VAD, devices, covering/stabilization and nursing records. The questionnaire was prepared by researchers based on the recommendations of the Infusion Therapy Standards of Practice ^(^ 7 ^)^ and evaluated by three judges with expertise in intravenous therapy. The reliability of the instrument was verified using Cronbach’s alpha, with a value equal to 0.82, demonstrating almost perfect internal consistency. 

 The questionnaires were sent to participants via e-mail, direct mail (Zievo ^®^ ), social networks (Instagram ^®^ and/or Facebook ^®^ ), WhatsApp ^®^ and websites of the *Conselhos Regionais de Enfermagem* (COREN) in some regions of Brazil. The submission was managed using the Research Electronic Data Capture (REDCap ^®^ ) tool, made available by a federal educational institution in the state of São Paulo. 

### Data analysis

A descriptive analysis of the data was carried out, with the categorical variables described using absolute and relative frequencies. To compare the frequencies of the qualitative variables of the three groups, the Chi-square test was used for k independent samples, considering a significance level of 5% and a 95% confidence interval (CI). For variables with an expected frequency < 20%, the likelihood ratio test was used.

Adjusted residual analysis (> 1.96 or < -1.96) was applied to identify the categories that showed the greatest difference between the expected counts and actual counts in relation to the sample size. Positive residues indicated an observed frequency greater than expected and negative residues, the opposite.

The p value was calculated in OpenEpi version 3.0, using the Table L x C calculations option, and data analysis was performed in the Statistical Package for the Social Sciences (SPSS), version 26.0.

### Ethical aspects

 The research met the assumptions of Resolution 466/12 and was approved by the Research Ethics Committee (CEP, acronym in Portuguese) of the *Universidade Federal de São Paulo* under CAAE n.º 79646317.7.0000.5505 and opinion n.º 3.274.729. The free and informed consent form containing information about the research was sent by REDCap ^®^ . 

## Results

1,214 (41%) nurses, 1,166 (39.4%) technicians and 204 (6.9%) nursing assistants participated, totaling 2,960 participants. Of this number, 376 (12.7%) were excluded for not declaring their professional category, resulting in a final sample of 2,584 participants. The majority were women living in the Southeast macro-region. The average age of nurses was 39.52 (± 8.74) years old, of technicians 39.66 (± 9.22) years old, and of nursing assistants, 40.61 (± 10.57) years old.

 In Table 1 , it can be seen that most professionals do not explain the PIC procedure to the family member and/or guardian or to adult patients, but they use different strategies to prepare the pediatric patient before PIC, as well as resources for managing the pain. 

 Almost all respondents do not clean their hands before insertion of the VAD, and a little more than half of nurses and nursing assistants change their procedural gloves before attempting PIC. In the group of nursing technicians, this distribution was more equitable. Furthermore, a considerable number of Nursing professionals do not use new antisepsis material with each PIC attempt, nor a new device ( Table 1 ). 

**Table 1:** - Patient preparation and care performed by Nursing professionals before each attempt to insert the vascular access device according to professional category (n = 2,584). Brazil, 2022

**Variable**	**Nurse (n= 1,214)**		**Nursing technician (n= 1,166)**		**Nursing assistant (n= 204)**		**p-value**
	**N**	**%**	**N**	**%**	**N**	**%**	
**Explanation of the procedure to the family member/guardian**							
Yes	323	26.6 *	438	37.6 ^†^	75	36.8	
No	891	73.4 ^†^	728	62.4 *	129	63.2	**0.000** ^‡^
**Explanation of the procedure to the patient**							
Yes	92	7.6	73	6.3	7	3.4 *	
No	112	92.4	1093	93.7	197	96.6	0.068 ^‡^
**Child preparation strategy** ^§^							
Therapeutic toy	186	17.9 ^†^	115	20.9 *	17	20.7	
Booklets	45	4.3 ^†^	22	4.0 *	5	6.1	
Virtual reality	42	4.0	30	5.4	4	4.9	
Breastfeeding	131	12.6 *	54	9.8 ^†^	7	8.5 ^†^	
Skin-to-skin contact	185	17.8 ^†^	116	21.1 *	15	18.3 *	
Winding	201	19.3 ^†^	83	15.1 *	13	15.8 *	
Sweet solution	148	14.2 ^†^	45	8.2 *	4	4.9 ^*^	
Not used	102	9.8	85	15.4	17	20.7	**0.000** ^||^
**Use of resources for pain management before catheterization**							
Yes	749	62.7	657	58.3 *	134	67.0	
No	446	37.3	470	41.7	66	33.0	**0.019** ^‡^
**Hand hygiene**							
Yes	7	0.6	2	0.2	-	-	
No	119	99.4	1144	99.8	201	100	0.123 ^||^
**Change of gloves after each PIC** ^¶^ **attempt**							
Yes	775	64.4 ^†^	571	49.9	112	56.0	
No	429	35.6	573	50.1 ^†^	88	44.0	**0.000** ^‡^
**Use of new material for antisepsis**							
Yes	133	11.1 ^†^	75	6.5	8	4.0	
No	107	88.9	1071	93.5 ^†^	192	96.0 ^†^	**0.000** ^‡^
**Use of a new device**							
Yes	114	9.5 ^†^	66	5.8 *	18	9.0	
No	108	90.5 *	1078	94.2 ^†^	183	91.0	**0.003** ^‡^

* Adjusted residual < -1.96

† Adjusted residual > 1.96

‡ Pearson’s Chi-square test for k independent samples

§ Question that allowed multiple answers

|| Likelihood ratio test

¶ PIC = Peripheral intravenous catheterization

 The most cited criteria for selecting peripheral veins were the time of use of peripheral intravenous therapy and the caliber of the device. Regarding the preferred site for venous catheterization in children and adults, Nursing professionals highlighted hands, arms and forearms ( Table 2 ). 

 Most nurses and technicians evaluate the venous network through heat application, while nursing assistants use traditional methods, such as palpation and visualization of the vessel. Most Nursing professionals use clinical tools to evaluate difficult venous networks ( Table 2 ). 

 There were statistically significant differences between professionals who evaluated the venous network using venoscope, vascular ultrasound or heat application, tools for evaluating difficult venous network, and who listed the time of use of IVT, the classification and nature of the medication and the visibility of the vein as criteria for selection of peripheral veins. In the variable catheterization site in children and adults, considerable differences were also observed between the three groups, according to the adjusted residual values presented in Table 2 . 

 The most frequently cited tourniquet technique was the universal tourniquet, with time varying between 30 seconds and 1 minute. Regarding the distance between the tourniquet and the catheterization area, there was an equitable distribution. It is also noted that a significant number of Nursing professionals use the direct method of device insertion and make two or three catheterization attempts ( Table 3 ). 

**Table 2:** - Evaluation of the venous network and selection of peripheral veins according to professional category (n = 2,584). Brazil, 2022

**Variable**	**Nurse** **(n= 1,214)**		**Nursing technician (n= 1,166)**		**Nursing assistant** **(n= 204)**		**p-value**
	**N**	**%**	**n**	**%**	**n**	**%**	
**Method for evaluating the venous network** *							
Traditional method	54	11.5	69	21.9	12	30.0	
Venoscope ^®^	67 ^†^	14.2	42 ^†^	13.3	1 ^†^	2.5	
Accuvein ^®^	5	1.1	7	2.2	1	2.5	
Vein Viewer ^®^	45	9.6	29	9.2	2	5.0	
Vascular ultrasound	71 ^†^	15.6	22 ^‡^	7.0	2 ^‡^	5.0	
Heat application	150 ^†^	31.9	81 ^‡^	25.7	11 ^‡^	27.5	
Double tourniquet	46	9.8	30	9.5	3	7.5	
Triple tourniquet	7	1.8	3	0.95	1	2.5	
Not used	25	5.3	32	10.2	7	17.5	**0.000** ^§^
**Use of a clinical tool to evaluate difficult venous networks**							
Yes	1069 ^†^	88.8	964 ^‡^	84.5	188 ^†^	92.5	
No	135	11.2	177	15.5	15	7.4	**0.000** ^||^
**Criteria for selecting** *							
Time of use of peripheral intravenous therapy	921 ^†^	19.9	700 ^‡^	18.8	116 ^‡^	19.2	
Medications classification	780 ^†^	16.9	485 ^‡|^	13.0	70 ^‡^	11.6	
Nature of the medication	442 ^†^	9.6	234 ^‡^	6.3	38 ^‡^	6.3	
Device caliber	796	17.2	753	20.2	128	21.2	
Vein visibility	650 ^†^	14.1	570 ^‡^	15.3	89 ^‡^	14.7	
Vein palpability	700	15.1	694	18.6	114	18.9	
Patient preference	332	7.2	286	7.7	49	8.1	**0.000** ^||^
Site of venous catheterization in children *							
Hand	550	26.0	510	26.9	83	27.3	
Arm	378	17.8	390	20.1	65	21.4	
Forearm	514 ^†^	24.3	455	24.0	63 ^‡^	21.0	
Foot	190 ^†^	9.0	139 ^‡^	7.3	14 ^‡^	4.6	
Ankle	35 ^†^	1.6	16 ^‡^	0.8	3	0.9	
Head	46 ^†^	2.2	18 ^‡^	0.9	2	0.6	
Neck	38 ^†^	1.8	5 ^‡^	0.3	1	0.3	
Does not perform the procedure on children	366	17.3	363	19.1	73	24.0	**0.000** ^§^
Site of venous catheterization in adults *							
Hand	631 ^‡^	25.3	685 ^†^	27.7	113	26.9	
Arm	687 ^‡^	27.5	777 ^†^	31.4	140 ^†^	33.3	
Forearm	941	37.7	916	37.0	154	36.7	
Foot	23	0.9	39	1.6	5	1.2	
Ankle	5	0.2	21 ^†^	0.8	1	0.2	
Head	4	0.16	1	0.04	1	0.2	
Neck	145 ^†^	5.8	27 ^‡^	1.1	5 ^‡^	1.2	
Does not perform the procedure on adults	60 ^†^	2.4	9 ^‡^	0.4	1 ^‡^	0.2	**0.000** ^§^

* Question that allowed multiple answers

† Adjusted residual > 1.96

‡ Adjusted residual < -1.96

§ Likelihood ratio test

|| Pearson’s Chi-square test for k independent samples

 The most cited VADs were those with a safety device. Regarding the material of these devices, nurses mentioned polyurethane and Teflon ^®^ , and in the group of nursing technicians and assistants more than half were unable to inform the constitution of the devices used in their practices ( Table 4 ). 

 Most professionals use the 24 Gauge (G) device in children and the 20G device in adults. It can also be seen that more than one criterion is adopted for device selection, with the most listed being: vessel caliber, skin fragility, duration of therapy use and type of infusion ( Table 4 ). 

 About materials for covering and stabilizing the VAD, Micropore ^®^ type adhesive tape was frequently mentioned by Nursing professionals, followed by transparent film. Referring to the identification of the PIC, it was found that less than half of the professionals indicate the record of the time, caliber and name of the professional responsible for the procedure ( Table 4 ). 

 There were differences in the group of nurses and nursing technicians who used all types of device materials, VAD with 22G caliber in adults and transparent cover. Among the assistants, the difference was found in the Teflon ^®^ and polyurethane categories, the group that did not know the device number and that used IV-Fix ^®^ ( Table 4 ). 

**Table 3:** - Technique and procedures adopted for insertion of a peripheral vascular device by Nursing professionals performing peripheral intravenous catheterization (n = 2,584). Brazil, 2022

**Variable**	**Nurse** **(n= 1,214)**		**Nursing technician (n= 1,166)**		**Nursing assistant** **(n= 204)**		**p-value**
	**N**	**%**	**n**	**%**	**n**	**%**	
**Technique for limb tourniquet** *							
Single-use tourniquet	273 ^†^	17.6	355 ^‡^	24.8	68 ^‡^	26.4	
Universal tourniquet	675 ^‡^	43.4	594 ^†^	41.6	118	45.9	
Glove or part of the glove for procedure	514 ^‡^	33.1	426 ^†^	29.8	69	26.8	
Professional’s hands	92 ^‡^	5.9	53 ^†^	3.7	2 ^†^	0.8	**0.000** ^§^
**Tourniquet time**							
30 sec	425	35.3	380	33.2	59	29.2	
1 minute	397	33.0	403	35.2	78	38.6	
1 min 30 sec	69	5.7	91	7.9	11	5.4	
2 minutes	62	5.2	63	5.5	8	4.0	
Does not control time	250	20.8	209	18.2	46	22.8	0.126 ^||^
**Tourniquet-catheterization area distance**							
As close to the site as possible	582 ^‡^	48.5	502 ^†^	43.9	86	43.7	
As far away from the site as possible	516	43.0	508	44.4	84	42.6	
Indifferent	103 ^†^	8.6	133 ^‡^	11.6	27	13.7	**0.028** ^||^
**Device insertion method**							
Direct	959 ^‡^	80.5	863 ^†^	76.1	143 ^†^	73.0	
Indirect	200	16.8	218	19.2	38	19.4	
Does not know	33 ^†^	2.8	53	4.7	15 ^‡^	7.7	**0.002** ^||^
**Maximum catheterization attempts**							
1 attempt	20 ^†^	1.7	49 ^‡^	4.3	11 ^‡^	5.5	
2 attempts	597 ^†^	50.0	620 ^‡^	54.2	113	56.8	
3 attempts	427	35.7	388	33.9	56 ^†^	28.1	
4 attempts	74 ^‡^	6.2	33 ^†^	2.9	7	3.5	
As many as necessary	77 ^‡^	6.4	53 ^†^	4.6	12	6.0	**0.000** ^||^

* Question that allowed multiple answers

† Adjusted residual > 1.96

‡ Adjusted residual < -1.96

§ Likelihood ratio test

|| Pearson’s Chi-square test for k independent samples

**Table 4:** - Devices, covering/stabilization and nursing records carried out by Nursing professionals performing peripheral intravenous catheterization (n = 2,584). Brazil, 2022

**Variable**	**Nurse** **(n= 1,214)**		**Nursing technician** **(n= 1,166)**		**Nursing assistant** **(n= 204)**		**p-value** *
	**n**	**%**	**N**	**%**	**N**	**%**	
**Device type** ^†^							
Simple	508	34.8	501	35.4	101	40.0	
With safety device	944	64.7	907	64.0	150	59.2	
Does not know	7	0.5	8	0.6	2	0.8	0.560
**Device material** ^†^							
Teflon ^®^	393 ^‡^	28.4	231 ^§^	20.9	41 ^§^	21.6	
Polyurethane	421 ^‡^	30.5	149 ^§^	13.5	16 ^§^	8.4	
Vialon ^®^	62 ^‡^	4,5	30 ^§^	2,7	7	3,7	
Does not know	506	36,6	695	62,9	126	66,3	**0,000**
**Device caliber in child** ^†^							
18 Gauge	33	2,0	44	2,9	7	2,8	
20 Gauge	79	4,9	101	6,6	14	5,5	
22 Gauge	440	27,1	390	25,6	58	23,0	
24 Gauge	831	51,1	777	51,1	130	51,6	
Does not know	242	14,9	209	13,7	43	17,1	0,239
**Device caliber in adult** ^†^							
18 Gauge	316	15.6	314	15.7	50	14.4	
20 Gauge	839	41.3	774	38.6	131	37.9	
22 Gauge	637 ^§^	31.4	708 ^‡^	35.3	111	32.1	
24 Gauge	168	8.3	172	8.6	36	10.4	
Does not know	70 ^‡^	3.4	37 ^§^	1.8	18 ^‡^	5.2	**0.001**
**Criteria for selecting the device** ^†^							
Largest available	29	0.9	25	0.9	3	0.6	
Minor available	64 ^‡^	1.9	38	1.3	2	0.4	
Vessel caliber	996	30.4	899	31.6	151	31.4	
Skin fragility	704	21.5	617	21.7	108	22.5	
Therapy use time	737	22.5	656	23.1	109	22.7	
Type of infusion	743	22.7	605	21.3	107	22.3	0.358
**Material used for covering/stabilization** ^†^							
Adhesive type adhesive tape	318	14.9	298	15.1	52	15.6	
Micropore ^®^ type adhesive tape		28.9	600	30.5	119	35.6	
Transparent film	529 ^‡^	24.8	419 ^§^	21.3	75	22.4	
IV-Fix ^®^	279	13.1	270	13.7	28 ^§^	8.4	
Tegaderm ^®^	387	18.2	382	19.4	60	17.9	**0.026**
**Identification of peripheral intravenous catheterization** ^†^							
Procedure date	94	7.9	88	8.2	19	9.3	
Procedure time	454	38.1	408	37.9	65	31.7	
Device caliber	399	33.5	325	30.2	68	33.2	
Responsible professional	243	20.4	254	23.6	53	25.8	0.214

* Pearson’s Chi-square test for k independent samples

† Question that allowed multiple answers

‡ Adjusted residual > 1.96

§ Adjusted residual < -1.96

## Discussion

In this study, significant variations were found in practices related to PIC implemented by nurses, technicians and nursing assistants working in the geographic regions of the country, some in accordance with current recommendations, while others are not in line with the best practices for PIC.

 It was observed that nurses are the professionals who least advise family members/guardians about the PIC procedure. In this context, it is important to highlight that care planning in IVT must involve the family, as they collaborate in the process of treatment and health recovery of their members ^(^ 12 ^)^ . This relationship must be based on a partnership that respects the patient’s values, preferences, feelings and needs ^(^ 13 ^)^ . 

 In this sense, providing the family member/guardian and/or patient with information about the correct identification of these events helps to avoid the suffering and discomfort caused by new attempts at PIC, which can result in depletion of venous access, fear of needles and hospital avoidance ^(^ 14 ^)^ . 

 In terms of “preparing pediatric patients before PIC”, in general, the Nursing professionals in this study adopt more than one strategy, in addition to the attention given to pain management before catheterization. Cross-sectional research carried out in the Neonatal Intensive Care Unit of a hospital in Fortaleza-CE showed behavioral and physiological changes in newborns undergoing PIC and who did not use non-pharmacological measures to relieve pain ^(^ 15 ^)^ . 

 Venous catheterization is a painful procedure that brings an unpleasant sensory and emotional experience ^(^ 16 ^)^ . Therefore, the Nursing team must make it less stressful, through strategies for controlling pain and applying instruments to measure the pain experienced by the patient ^(^ 17 ^)^ . 

In the present research, aseptic care before each attempt to insert the VAD contradicts globally recognized practice standards, such as non-hygiene of hands, non-use of new antisepsis material or a new device. Regarding changing procedure gloves, an improvement in percentages is observed, but it highlights that professionals may be replacing hand hygiene with the use of gloves. In the group of nurses, this rate was lower, but still represents a notable number of professionals.

 Hand hygiene cannot be replaced by the use of procedure gloves. Professionals justify this change by claiming that there is a loss of touch when palpating the vein or PIC, and many use the glove only when connecting the VAD to the equipment or syringe ^(^ 18 ^)^ . Several surveys attest that professionals’ adherence remains low, even with so many recommendations on the relevance of hygiene practices before insertion of the VAD. 

 Therefore, looking at the Theory of Planned Behavior (TPB) can shed light on this and other issues related to PIC. This is a predictive model of behavior in which three psychological constructs (attitude, subjective norm and perception of control) explain that the intention of professionals is the immediate antecedent of behavior ^(^ 19 ^)^ . 

 Attitude links personal interest in carrying out a certain behavior to the results expected from it. The Nursing professional, based on their behavioral beliefs, evaluates the consequences of not adopting correct PIC practices, and the result of this analysis will determine their intention ^(^ 19 ^)^ . 

 The subjective norm concerns the professional’s perception of social pressure to perform a certain behavior ^(^ 19 ^)^ . In many work contexts, the social pressure on nurses for the team to carry out aseptic procedures before each PIC attempt is non-existent, and they themselves do not adopt such a stance. 

 The perception of control, in turn, demonstrates the degree to which the professional feels capable of carrying out a behavior, and can be influenced by attitude and subjective norms. Even if the Nursing professional is in favor of adopting appropriate PIC practices, an individual may succumb to social pressure exerted by other colleagues or to low control ^(^ 19 ^)^ . 

 Regarding the assessment of the venous network, professionals report the use of more than one criterion. The traditional method and heat application were the most cited by nursing technicians and assistants, while nurses highlight the use of technologies such as vascular ultrasound and Venoscope ^®^ . 

 In the traditional method, professionals are guided by anatomical reference points, inspection and palpation of the peripheral vein. Despite being commonly used in clinical practice by secondary level and technical professionals, a systematic review study found the superiority of the use of ultrasound in relation to traditional catheterization ^(^ 20 ^)^ . This data highlights that vein visualization technology allows greater assertiveness, agility and safety, fewer attempts for successful catheterization, fewer complications, reduced procedure time and greater patient satisfaction ^(^ 21 ^)^ . 

 A worrying result is that some nursing technicians and assistants claim to use vascular ultrasound in their professional practices, despite the use of this technology being exclusive to nurses. Furthermore, the culture that PIC is a simple procedure that can be performed by any member of the Nursing team still prevails ^(^ 22 ^)^ . 

 Therefore, the nurse needs to assume legal responsibility for inserting the peripheral device, as, associated with the complexity of the procedure, there are conditions that make peripheral venipuncture difficult (DPVP), such as premature, malnourished, obese or chronically ill children and adults with high care complexity ^(^ 23 ^)^ . In the latter case, the prevalence of DPVP is 59.3% and varies depending on the patient’s clinical condition ^(^ 24 ^)^ . 

Most Nursing professionals mentioned the use of clinical tools to evaluate the difficult venous network. Although the percentage was higher in the group of nursing assistants, it was observed that they did not know the definition of clinical tool, as they listed the use of anatomy, technology, tourniquet, device, among others, as synonyms.

 The adoption of measurement instruments in clinical practice, such as scales, protocols with recommendations, Bundles and flowcharts to assess the difficulty of PIC can contribute to the quality of nursing care, with a consequent reduction in the failure rate and depletion of blood vessels, as vascular care is important for preserving the health of vessels throughout an individual’s life ^(^ 25 ^)^ . 

Other important precautions include the selection of devices of appropriate caliber, the choice of the site of peripheral veins that support the therapy to be implemented, the VAD insertion technique, and the administration of solutions and drugs in appropriate quantities and concentrations.

 Several criteria for the selection of peripheral veins were highlighted by professionals in this study. The time of use of peripheral IVT and the caliber of the device were cited by all professional categories. The nurses also listed the classification of the medication, while the nursing technicians and assistants added the palpability of the vein. The type of solution to be infused, the infusion time and the condition of the veins must also be considered ^(^ 26 ^)^ during the treatment of hospitalized patients. 

Additionally, the Nursing team must monitor the effects and know the nature of the medications, in order to avoid local and/or systemic complications. In the present study, this criterion was little considered by Nursing professionals.

Another relevant aspect rarely mentioned by the Nursing team concerns patient preference. Perhaps verbal communication between professional and patient before VAD insertion is not routine within the health service.

 In the present study, Nursing professionals mentioned hands, arms and forearms as the main sites of choice for venous catheterization in children. Descriptive study developed in Neonatal Intensive Care Units (NICU) and Pediatric Intensive Care Units (PICU) showed that 55.5% of Nursing professionals in the NICU and 34.6% in the PICU chose the veins on the back of the hands as the first option for PIC ^(^ 27 ^)^ . 

 These data are in accordance with the American Infusion Nurses Society (INS), which recommends choosing the vessels with the greatest chance of lasting the entire prescribed therapy, such as the upper limbs and the back of the hands of pediatric patients, avoiding areas of flexion, so as not to limit the child’s movement ^(^ 7 ^)^ . 

 In contrast, INS Brazil states that the first PIC attempt should be initiated in the most distal region, considering the particularities of each drug and possible complications ^(^ 28 ^)^ . It also recommends the choice of head veins in children under three years of age and, if they cannot walk, the use of foot veins ^(^ 7 ^)^ . 

 Global cross-sectional research carried out in 278 hospitals in 47 countries with data from 4,206 children identified the hand (51%; n= 2143) as the most accessed site for VAD placement. However, in North America, Australia and New Zealand, the antecubital fossa (rate varying between 21.4 and 24.5%) was the most mentioned region ^(^ 29 ^)^ . 

 In emergency care, this region is frequently accessed due to the possibility of rapid infusion of large volumes and, for this reason, some authors recommend this site because of the larger caliber of the veins ^(^ 12 ^)^ . However, others do not recommend, due to the limitation of mobility of the patient’s limb, unless flexible venous devices are used or this is the only venous access available ^(^ 26 ^)^ . 

 In adults, the cephalic and basilic veins in the forearm are the preferred sites for PIC. Most of the professionals interviewed mentioned the arm and forearm, but some chose the lower limbs as the catheterization site. This finding, despite the low percentage, causes concern, due to the greater risk of embolism, thrombophlebitis and infection resulting from PIC in leg/foot veins in adults ^(^ 26 ^)^ . 

After vein selection, techniques and procedures are adopted to facilitate VAD insertion, such as the use of a tourniquet, which allows venodilation and facilitates visualization and the PIC process. Regarding how long the tourniquet stays on the patient, almost all nurses reported that they leave it on for 30 seconds, while technicians and nursing assistants keep it on for up to 1 minute. However, a significant percentage of professionals do not control the time.

 Failure to control the time can cause diagnostic errors such as hemolysis, increased potassium levels and/or calcium levels, as well as generating complications during catheterization, such as bruising, tingling and, in extreme cases, Trousseau’s sign ^(^ 30 ^)^ . 

 There are also cases of forgotten tourniquets on patients’ extremities after attempts to place the VAD. Therefore, the nurse should be alert for signs of tourniquet retention, which include pain in the extremities, tingling, edema, poorly flowing intravenous infusion, leakage at the VAD insertion site and/or catheterization sites ^(^ 31 ^)^ . 

 Another predisposing factor for the occurrence of complications during IVT is the VAD insertion technique, whether direct or indirect. The professionals in this study frequently mentioned the use of the direct method, but a small percentage of nursing assistants and technicians did not know how to identify the device insertion method. Evidence indicates that the direct method is more associated with the occurrence of intravenous complications ^(^ 32 ^)^ . 

In view of this, health services have sought to guarantee care free from risks and damages. And in this context, nurses play a key role in assessing the risk of difficult peripheral venous access, in order to avoid numerous unsuccessful catheterization attempts.

 A study carried out in Portugal showed that nurses need two to eight catheterization attempts to successfully insert a VAD, with rates varying between 19.4% and 23.7% ^(^ 33 ^)^ . This number tends to increase to an average of five attempts per person, and can vary between one and 20 attempts when considering the entire period of treatment of the hospitalized patient ^(^ 33 ^)^ . 

 The *Agência Nacional de Vigilância Sanitária* (ANVISA) recommends up to a maximum of two attempts per professional with the aid of vein visualization technologies, and limits the number of attempts to a maximum of four punctures (two by different professionals) ^(^ 25 ^)^ . 

 Repeated PIC attempts negatively affect the overall patient experience and can cause vessel damage, increasing the chance of using central venous access devices ^(^ 3 ^)^ . The professionals participating in this research mentioned making three attempts, which already contradicts current regulations, with this rate being higher in the group of nurses. 

 Concerning practices related to device care and covering/stabilization, it was observed that the device materials most used by nurses were polyurethane and Teflon ^®^ . Some of the nursing technicians and assistants used a Teflon ^®^ device, and more than half were unable to inform the type of material (a considerable percentage of nurses also fall into this category). 

 Lack of knowledge about the constitution of the VAD increases the risk of complications, as evidence shows that devices made of polyurethane are associated with low incidences of infectious complications when compared to those made of polyvinylchloride and polyethylene ^(^ 34 ^)^ . 

In general, Nursing professionals use 24G devices for children and 20G for adults. It is noted that the choice of caliber in the pediatric population resulted in a considerable percentage of professionals who did not know the device number, especially nursing assistants and nurses.

 For pediatric patients, the use of 22G and 24G calibers is recommended, but a small percentage of respondents use 18G and 20G calibers. Devices with smaller caliber are associated with fewer complications, in addition to causing less mechanical aggression to the vein wall by the cannula and less obstruction of blood flow within the vessel ^(^ 9 ^)^ , but this statement must take into account the patient’s age and the characteristics of the venous network. 

Children’s veins are more fragile when compared to adults, therefore, assessing the risks and benefits of each type of device, such as caliber, constitution and selection criteria, is essential. In this last item, Nursing professionals, for the most part, chose the time of use of the therapy as the main criterion for selecting the device.

 After PIC, the data highlighted that the majority of Nursing professionals, including nurses, use Micropore ^®^ type adhesive tape to stabilize and cover the device. This finding is worrying, given that the use of non-sterile adhesive tapes is a practice widely observed in developing countries. 

 It is common to see numerous Nursing professionals who cut strips of tape before performing PIC and attach them to their own uniform, tray or patient’s bedside table. The purpose of the covering is to protect the VAD insertion site and reduce the risk of infection, therefore this routine practice contaminates the tape after opening the original packaging. Adhesive tape must be sterile and changed whenever it is damp, dirty, loose or its integrity is compromised. If contamination is suspected, exchange must be immediate ^(^ 7 ^)^ . 

Another significant aspect after insertion of the VAD is its identification, which allows communication between the Nursing team and enables continuity of care. In the present study, it is noted that the team is not concerned with recording the date of the procedure, as the percentages of adoption of this practice were very low, including in the group of nurses. Most professionals record the time of the procedure, the caliber of the device and the name of the responsible professional on the device.

 Failure to record the date may contribute to the high prevalence of idle catheters, defined as not used in the previous 24 hours and not planned for use in the next 24 hours. The literature indicates that around 14 to 50% of peripherals are kept on the patient “just in case” they are needed ^(^ 35 ^)^ . 

 In some institutions, the recommended length of stay for the VAD is 96 hours. For ANVISA, the change must not be less than 96 hours, and the routine assessment of the Nursing team will allow the decision to maintain the device for a longer period of time, or when clinically indicated ^(^ 9 ^)^ . 

Furthermore, the World Health Organization (WHO) adds that PIC registration is a patient identification requirement and a goal that guarantees safety in health services and mitigates the occurrence of errors.

Dating the catheterization allows monitoring of the VAD since its insertion, in addition to making it possible to check its validity in situations when removal is scheduled. And, in the event of a complication, the possibility of evaluating the reasons and taking action to avoid worsening.

The results of this study disseminate best practices related to peripheral VAD insertion, particularly with regard to care before each insertion attempt, which involves patient preparation, assessment of the venous network, techniques and procedures adopted, devices, covering/stabilization and nursing records.

As a limitation of the study, the choice of the epidemiological design that analyzed the practices in a specific manner stands out, which has little power to generate robust evidence about the PIC carried out by Nursing professionals. Furthermore, non-probability sampling interferes with the external validity of the study.

## Conclusion

Most Nursing professionals do not involve the patient and family in care, adopt strategies to prepare the patient before catheterization and do not maintain aseptic care. When evaluating the venous network, the percentage of professionals who did not use evaluation methods and/or clinical tools was much lower.

Many professionals do not control the appropriate tourniquet time and have difficulty identifying the correct distance between the tourniquet and the catheterization area. Furthermore, the most adopted device insertion method is precisely the one that is most associated with the occurrence of intravenous complications, and almost half of nurses and nursing technicians try to puncture the same patient three or more times.

 Regarding the characteristics of the device, professionals had similar knowledge about the type of device and caliber used in children and adults. There is a large number of nursing technicians and assistants who do not know the composition of the devices used in the work environment. Most professionals used Micropore ^®^ type adhesive tape to cover and stabilize the VAD, and almost all of the three professional categories investigated did not record the date of the PIC. 

In view of the results found, it is clear that nursing technicians and assistants are the professionals who least comply with what is recommended in recognized guidelines. However, nurses’ practice also presents deviations in relation to scientific evidence, and their performance was not very different from secondary/technical level professionals. Thus, weaknesses were revealed in the care offered to the patient before the insertion of the peripheral vascular access device that could compromise safety and cause complications.

It is understood that it is necessary to implement educational actions and theoretical-practical training of the Nursing team, including nurses. Therefore, technical-scientific knowledge can guarantee effectiveness in treatment and the quality of care provided.
