# Supplementary material for: National practice of Nursing professionals in the insertion of peripheral vascular access devices
Source: Rev Lat Am Enfermagem. 2024 Sep 23;32:e4314. doi: 10.1590/1518-8345.6673.4314 (PMC11421520; doi:10.1590/1518-8345.6673.4314)
Supplement: Translation

# Translation: Práctica nacional de los profesionales de Enfermería en la inserción de dispositivos de acceso vascular periférico *

**DOI:** 10.1590/1518-8345.6673.4313

**Published:** 2024-09-23

**Authors:** Bianka Sousa Martins Silva, Luciano Marques dos Santos, Patrícia Kuerten Rocha, Aline Nair Biaggio Mota, Ariane Ferreira Machado Avelar, Denise Miyuki Kusahara

**Affiliations:** 1 Universidade Federal de São Paulo, Escola Paulista de Enfermagem, São Paulo, SP, Brasil; 2 Universidade Estadual de Feira de Santana, Departamento de Saúde, Feira de Santana, BA, Brasil.; 3 Universidade Federal de Santa Catarina, Departamento de Saúde, Santa Catarina, SC, Brasil.; 4 BIOTRONIK Comercial Médica, Medical Affairs, São Paulo, SP, Brasil.

**Keywords:** Cateterismo Periférico, Infusiones Intravenosas, Dispositivos de Acceso Vascular, Equipo de Enfermería, Cuidado de Enfermera, Competencia Profesional

## Abstract

**Objetivo:**

identificar y comparar la práctica de los profesionales de Enfermería respecto a la inserción de dispositivos de acceso vascular periférico, según categoría profesional.

**Método:**

estudio descriptivo seccional realizado entre julio de 2021 y mayo de 2022 con 2.584 profesionales de Enfermería, mediante cuestionario validado por tres jueces con experiencia en terapia intravenosa, que contiene variables relacionadas al cateterismo y al dispositivo de acceso vascular. Se realizó análisis descriptivo e inferencial.

**Resultados:**

la mayoría de los profesionales no prepara al paciente ni realizan algunos cuidados esenciales antes de intentar el cateterismo intravenoso periférico. En cuanto al sitio preferencial para el cateterismo, destacan las manos, el brazo y el antebrazo. No hay control sobre el tiempo del torniquete, y el paciente es punzado más de tres veces. Los materiales del dispositivo más utilizados son el poliuretano y el Teflón ^®^ , se adopta más de un criterio para la selección del dispositivo, y la cinta adhesiva tipo Micropore ^®^ fue la cobertura más citada por los profesionales de Enfermería. La identificación del cateterismo no fue adecuada.

**Conclusión:**

los técnicos y auxiliares de enfermería son los profesionales que menos cumplen con lo recomendado en guías reconocidas. La práctica de los enfermeros también presenta desviaciones de la evidencia científica.

## Puntos destacados

**(1)** La mayoría de los profesionales de Enfermería no involucra al paciente y a su familia en el cuidado.

**(2)** Los técnicos/auxiliares son los que menos cumplen con los estándares recomendados.

**(3)** Los enfermeros también presentan desviaciones en relación a la evidencia científica.

**(4)** El desempeño del enfermero no fue muy diferente al de los profesionales de nivel secundario.

**(5)** Existen debilidades en el cuidado que se ofrece al paciente.

## Introducción

 El cateterismo intravenoso periférico (CIP) se realiza ampliamente en pacientes en los más diversos entornos de atención a la salud, principalmente para la inserción de dispositivos de acceso vascular (DAV). Este procedimiento permite la implementación de diversas terapias, como la administración de líquidos, medicamentos, componentes sanguíneos y nutrición directamente al sistema circulatorio ^(^ 1 ^)^ . 

 Una investigación transversal multinacional realizada en cinco países de América Latina indica que alrededor del 70% de los pacientes hospitalizados se someten a CIP ^(^ 2 ^)^ . Aunque rutinaria, esta práctica puede causar complicaciones que resultan en morbimortalidad, aumento del tiempo de hospitalización y costos importantes que impactan el estado clínico y la evolución del paciente ^(^ 3 ^)^ . La (re)inserción del DAV es un procedimiento estresante para el paciente y su familia durante la hospitalización, y se asocia con una mayor fobia a las agujas y resistencia entre los adultos a la hora de buscar atención en los servicios de salud ^(^ 4 ^-^ 5 ^)^ . 

 La complejidad del CIP y su alta tasa de complicaciones asociadas indican que el enfermero debería ser el protagonista y actuar en todas las etapas del proceso, desde la selección del tipo, la instalación, hasta la retirada del DAV, en línea con recomendaciones y directrices internacionales reconocidas para la práctica de Enfermería ^(^ 6 ^-^ 7 ^)^ . 

 En el ámbito laboral, todos los profesionales de Enfermería que insertan DAV periférico deben estar capacitados de manera efectiva, para brindar a los pacientes un cuidado de alta calidad guiado por la mejor evidencia ^(^ 8 ^)^ . 

 Conocer las prácticas de inserción de DAV periféricos realizadas por los profesionales de Enfermería es imperativo para garantizar la efectividad del tratamiento y del cuidado brindado, además de evitar la aparición de complicaciones relacionadas con la terapia intravenosa (TIV). Evidencias indican que en el período comprendido entre junio de 2021 y mayo de 2022 se notificaron en los servicios de salud alrededor de 39.994 eventos adversos/complicaciones relacionados con dispositivos de acceso vascular ^(^ 9 ^)^ . 

Así, considerando la importancia del CIP y la necesidad de evaluar la ejecución de este procedimiento, con el propósito de mejorar la calidad de la atención, el objetivo de este estudio es identificar y comparar la práctica de los profesionales de Enfermería respecto a la inserción de dispositivos de acceso vascular periférico, según categoría profesional.

## Método

### Diseño del estudio

 Se trata de un estudio seccional del tipo *survey* descriptivo. Para descripción e informe del estudio se utilizó como referencia el *Strengthening the Reporting of Observational Studies in Epidemiology* (STROBE). 

### Lugar

Realizado en las cinco macrorregiones de Brasil.

### Período

La recolección de datos se realizó entre julio de 2021 y mayo de 2022.

### Población y criterio de selección

 La población del estudio fueron enfermeros, técnicos y auxiliares de enfermería residentes en las cinco macrorregiones de Brasil. Datos del *Conselho Federal de Enfermagem* (Cofen) (2022) registraron un total de 2.513.428 profesionales de Enfermería, de los cuales 613.827 (24,42%) eran enfermeros, 1.463.072 (58,21%) técnicos de enfermería y 436.529 (17,0%) auxiliares de enfermería ^(^ 10 ^)^ . 

Se incluyeron profesionales que actuaron en el cuidado directo de paciente sometido a CIP y que realizaban este procedimiento durante sus actividades habituales. No se incluyeron profesionales con hasta un año de finalizar su carrera de grado o curso técnico y sin experiencia previa en CIP, y se excluyeron aquellos que no informaron su categoría profesional en el cuestionario.

### Muestra

 Para el cálculo de la muestra se consideró una población de 2.513.428 profesionales de Enfermería, con una precisión del 3% y una frecuencia del 80% de CIP. La muestra mínima se estimó en 837 participantes. El cálculo del tamaño de la muestra se realizó mediante la herramienta *OpenEpi* , disponible de forma gratuita en internet ( https://www.openepi.com/Menu/OE_Menu.htm ). 

 Inicialmente, se envió un mensaje de correo electrónico a Cofen solicitando acceso a información de enfermeros pediátricos en Brasil. La solicitud generó el Proceso Administrativo COFEN N° 0940/2017, el cual fue remitido al Sector de Procuraduría General. Este emitió el dictamen legal nº 10/2018, a favor de proporcionar los datos luego de formalizar un Acuerdo de Compromiso entre el Cofen y la *Universidade Federal de São Paulo/Programa de Pós-Graduação em Enfermagem* . 

 Cofen puso a disposición una base de datos que contiene 613.987 registros activos de enfermeros y técnicos de enfermería. Las únicas informaciones presentes fueron la “categoría profesional” y el “correo electrónico ( *e-mail* )”, lo que dificultó la identificación del enfermero pediátrico. Por esta razón, se amplió la población para incluir a niños y adultos. 

 Además, también hubo muchos datos duplicados e inconsistencias, como que el mismo correo electrónico se repitió unas 16.644 veces (2,71%). De esta forma, se utilizó una muestra no probabilística intencional, que incluyó una fase de cadenas de referencia o bola de nieve ^(^ 11 ^)^ . Esta técnica se utilizó de forma complementaria, debido a que los datos del Cofen no representaban a la población de Enfermería. 

 El primer paso del método de muestreo de bola de nieve fue encontrar individuos pertenecientes a la población objetivo del estudio, a través de redes sociales ( *Instagram* ^®^ , *Facebook* ^®^ ) y *WhatsApp* ^®^ , grupos de investigación y profesionales de contacto de los investigadores. 

 Estos profesionales fueron denominados semilla de la muestra, y dieron origen a otros profesionales muestreados. A partir de la semilla, comenzó el proceso de bola de nieve, en el que los primeros profesionales fueron considerados ola cero. Los profesionales señalados por la ola cero que formaban parte de la población objetivo y que no formaban parte de la ola cero constituyeron la ola uno. A estas personas se les pidió que enviaran el *link* de la investigación a otros profesionales, y así sucesivamente. 

### Variables del estudio

Se investigaron variables relacionadas con el profesional (categoría profesional, tiempo de formación y número de DAV periférico insertado en un turno de trabajo de 12 horas), variables relacionadas con el CIP (explica el procedimiento al familiar/tutor y al paciente, estrategia y preparación del paciente, recurso para el manejo del dolor antes del cateterismo), variables relacionadas con los intentos de CIP (desinfecta las manos, cambia los guantes después de cada intento, utiliza material nuevo para la antisepsia en cada intento de cateterismo, cambia el dispositivo con cada intento de CIP), variables relacionadas con la inserción del DAV (sitio de cateterismo venoso en niños y adultos, método de inserción del dispositivo, método para evaluar la red venosa, uso de herramienta clínica para evaluar redes venosas difíciles, criterios para seleccionar venas periféricas, número de intentos de cateterismo, torniquete en extremidades, tiempo de torniquete y distancia del torniquete del área de cateterismo), variables relacionadas con el dispositivo y la cobertura (tipo y material del dispositivo, calibre del dispositivo utilizado en niños y adultos, criterios para la selección del dispositivo y material utilizado para la cobertura/estabilización del dispositivo) y variables relacionadas con el registro (identificación del CIP).

### Recolección de datos e instrumentos

 La recolección de datos se realizó mediante cuestionario que contenía preguntas sobre preparación del paciente, cuidados realizados por los profesionales de Enfermería antes del CIP, método de evaluación y selección de venas periféricas, técnica y procedimientos adoptados para la inserción del DAV, dispositivos, cobertura/estabilización y registros de enfermería. El cuestionario fue elaborado por los investigadores con base en las recomendaciones de los *Infusion Therapy Standards of Practice* ^(^ 7 ^)^ y evaluado por tres jueces con experiencia en terapia intravenosa. La confiabilidad del instrumento fue verificada mediante el alfa de Cronbach, con valor igual a 0,82, demostrando consistencia interna casi perfecta. 

 Los cuestionarios fueron enviados a los participantes por correo electrónico, correo directo ( *Zievo* ^®^ ), redes sociales ( *Instagram* ^®^ y/o *Facebook* ^®^ ), *WhatsApp* ^®^ y sitios *web* de los *Conselhos Regionais de Enfermagem* (COREN) de algunas regiones de Brasil. El envío fue gestionado mediante la herramienta *Research Electronic Data Capture* ( *REDCap* ^®^ ), puesta a disposición por una institución educativa federal del estado de São Paulo. 

### Análisis de los datos

Se realizó un análisis descriptivo de los datos, describiéndose las variables categóricas mediante frecuencias absolutas y relativas. Para comparar las frecuencias de las variables cualitativas de los tres grupos se utilizó la prueba Chi-cuadrado para k muestras independientes, considerando un nivel de significancia del 5% y un intervalo de confianza (IC) del 95%. Para variables con una frecuencia esperada < 20% se utilizó la prueba de razón de verosimilitud.

Se aplicó un análisis residual ajustado (> 1,96 o < -1,96) para identificar las categorías que mostraron la mayor diferencia entre los recuentos esperados y los recuentos reales en relación con el tamaño de la muestra. Los residuos positivos indicaron una frecuencia observada mayor a la esperada y los residuos negativos, lo contrario.

 El valor de p se calculó en *OpenEpi* versión 3.0, utilizando la opción de cálculos Tabla L x C, y el análisis de los datos se realizó en el *Statistical Package for the Social Sciences* (SPSS), versión 26.0. 

### Aspectos éticos

 La investigación cumplió con los supuestos de la Resolución 466/12 y fue aprobada por el Comité de Ética en Investigación (CEP, sigla en portugués) de la *Universidade Federal de São Paulo* con CAAE n.º 79646317.7.0000.5505 y dictamen n.º 3.274.729. El formulario de consentimiento libre e informado que contiene información sobre la investigación fue enviado por *REDCap* ^®^ . 

## Resultados

Participaron 1.214 (41%) enfermeros, 1.166 (39,4%) técnicos y 204 (6,9%) auxiliares de enfermería, totalizando 2.960 participantes. De ellos, 376 (12,7%) fueron excluidos por no declarar su categoría profesional, resultando una muestra final de 2.584 participantes. La mayoría eran mujeres residentes en la macrorregión Sudeste. La edad promedio de los enfermeros fue de 39,52 (± 8,74) años, de los técnicos 39,66 (± 9,22) años, y de los auxiliares de enfermería 40,61 (± 10,57) años.

 En la Tabla 1 , se observa que la mayoría de los profesionales no explica el procedimiento del CIP al familiar y/o tutor, ni a los pacientes adultos, pero utilizan diferentes estrategias para preparar al paciente pediátrico antes del CIP, así como recursos para el manejo del dolor. 

 Casi todos los encuestados no se limpian las manos antes de la inserción del DAV, y poco más de la mitad de los enfermeros y auxiliares de enfermería se cambian los guantes de procedimiento antes de intentar el CIP. En el grupo de técnicos de enfermería, esta distribución fue más equitativa. Además, un número considerable de profesionales de Enfermería no utilizan nuevo material de antisepsia en cada intento de CIP, ni un nuevo dispositivo ( Tabla 1 ). 

**Tabla 1:** - Preparación del paciente y cuidados realizados por profesionales de Enfermería antes de cada intento de inserción del dispositivo de acceso vascular según categoría profesional (n = 2.584). Brasil, 2022

**Variable**	**Enfermero** **(n= 1.214)**		**Técnico de enfermería** (n= 1.166)		**Auxiliar de enfermería** **(n= 204)**		**p-valor**			
	**n**	**%**	**N**	**%**	**n**	**%**	
**Explicación del procedimiento al familiar/tutor**							
Sí	323	26,6 *	438	37,6 ^†^	75	36,8	
No	891	73,4 ^†^	728	62,4 *	129	63,2	**0,000** ^‡^
**Explicación del procedimiento al paciente**							
Sí	92	7,6	73	6,3	7	3,4 *	
No	112	92,4	1093	93,7	197	96,6	0,068 ^‡^
**Estrategia de preparación infantil** ^§^							
Jugueteterapéutico	186	17,9 ^†^	115	20,9 *	17	20,7	
Folletos	45	4,3 ^†^	22	4,0 *	5	6,1	
Realidadvirtual	42	4,0	30	5,4	4	4,9	
Lactancia	131	12,6 *	54	9,8 ^†^	7	8,5 ^†^	
Contactopiel con piel	185	17,8 ^†^	116	21,1 *	15	18,3 *	
Devanado	201	19,3 ^†^	83	15,1 *	13	15,8 *	
Soluciónde sabor dulce	148	14,2 ^†^	45	8,2 *	4	4,9 *	
No utiliza	102	9,8	85	15,4	17	20,7	**0,000** ^||^
**Uso de recursos para el manejo del dolor antes del cateterismo**							
Sí	749	62,7	657	58,3 *	134	67,0	
No	446	37,3	470	41,7	66	33,0	**0,019** ^‡^
**Higienización de manos**							
Sí	7	0,6	2	0,2	-	-	
No	119	99,4	1144	99,8	201	100	0,123 ^||^
**Cambio de guantes después de cada intento de CIP** ^¶^							
Sí	775	64,4 ^†^	571	49,9	112	56,0	
No	429	35,6	573	50,1 ^†^	88	44,0	**0,000** ^‡^
**Uso de nuevo material para antisepsia**							
Sí	133	11,1 ^†^	75	6,5	8	4,0	
No	107	88,9	1071	93,5 ^†^	192	96,0 ^†^	**0,000** ^‡^
**Uso de nuevo dispositivo**							
Sí	114	9,5 ^†^	66	5,8 *	18	9,0	
No	108	90,5 ^*^	1078	94,2 ^†^	183	91,0	**0,003** ^‡^

* Residual ajustado < -1,96

† Residual ajustado > 1,96

‡ Prueba Chi-cuadrado de Pearson para k muestras independientes

§ Pregunta que permitía múltiples respuestas

|| Prueba de razón de verosimilitud

¶ CIP = Cateterismo intravenoso periférico

 Los criterios más citados para la selección de venas periféricas fueron el tiempo de uso de la terapia intravenosa periférica y el calibre del dispositivo. En cuanto al sitio preferido para el cateterismo venoso en niños y adultos, los profesionales de Enfermería destacaron manos, brazos y antebrazos ( Tabla 2 ). 

 La mayoría de enfermeros y técnicos evalúa la red venosa mediante la aplicación de calor, mientras que los auxiliares de enfermería utilizan métodos tradicionales, como palpación y visualización del vaso. La mayoría de los profesionales de Enfermería utiliza herramientas clínicas para evaluar redes venosas difíciles ( Tabla 2 ). 

 Hubo diferencias estadísticamente significativas entre los profesionales que evaluaban la red venosa mediante venoscopio, ecografía vascular o aplicación de calor, herramientas de evaluación de la red venosa difícil, y que mencionaron el tiempo de uso de la TIV, la clasificación y naturaleza de la medicación y la visibilidad de la vena como criterios para la selección de venas periféricas. En la variable sitio del cateterismo en niños y adultos también se observaron diferencias considerables entre los tres grupos, según los valores residuales ajustados presentados en la Tabla 2 . 

 La técnica de torniquete más citada fue el torniquete universal, con un tiempo que varió entre 30 segundos y 1 minuto. En cuanto a la distancia entre el torniquete y la zona de cateterismo hubo una distribución equitativa. También se observa que un número importante de profesionales de Enfermería utiliza el método directo de inserción del dispositivo y realiza dos o tres intentos de cateterismo ( Tabla 3 ). 

**Tabla 2:** - Evaluación de la red venosa y selección de venas periféricas según categoría profesional (n = 2.584). Brasil, 2022

**Variable**	**Enfermero** **(n= 1.214)**		**Técnico de enfermería** (n= 1.166)		**Auxiliar de enfermería** **(n= 204)**		**p-valor**
	**N**	**%**	**n**	**%**	**N**	**%**	
**Método para evaluación de la red venosa** *							
Método tradicional	54	11,5	69	21,9	12	30,0	
Venoscopio ^®^	67 ^†^	14,2	42 ^†^	13,3	1 ^†^	2,5	
*Accuvein* ^®^	5	1,1	7	2,2	1	2,5	
*Vein Viewer* ^®^	45	9,6	29	9,2	2	5,0	
Ecografía vascular	71 ^†^	15,6	22 ^‡^	7,0	2 ^‡^	5,0	
Aplicación de calor	150 ^†^	31,9	81 ^‡^	25,7	11 ^‡^	27,5	
Torniquete doble	46	9,8	30	9,5	3	7,5	
Torniquete triple	7	1,8	3	0,95	1	2,5	
No utiliza	25	5,3	32	10,2	7	17,5	**0,000** ^§^
**Uso de herramienta clínica para evaluación de red venosa difícil**							
Sí	1069 ^†^	88,8	964 ^‡^	84,5	188 ^†^	92,5	
No	135	11,2	177	15,5	15	7,4	**0,000** ^||^
**Criterios para selección de venas periféricas** *							
Tiempo de uso de la terapia intravenosa periférica							
Clasificación de la medicación	780 ^†^	16,9	485 ^‡^	13,0	70 ^‡^	11,6	
Naturaleza de la medicación	442 ^†^	9,6	234 ^‡^	6,3	38 ^‡^	6,3	
Calibre del dispositivo	796	17,2	753	20,2	128	21,2	
Visibilidad de la vena	650 ^†^	14,1	570 ^‡^	15,3	89 ^‡^	14,7	
Palpabilidad de la vena	700	15,1	694	18,6	114	18,9	
Preferencia del paciente	332	7,2	286	7,7	49	8,1	**0,000** ^||^
Sitio del cateterismo venoso en niños *							
Mano	550	26,0	510	26,9	83	27,3	
Brazo	378	17,8	390	20,1	65	21,4	
Antebrazo	514 ^†^	24,3	455	24,0	63 ^‡^	21,0	
Pie	190 ^†^	9,0	139 ^‡^	7,3	14 ^‡^	4,6	
Tobillo	35 ^†^	1,6	16 ^‡^	0,8	3	0,9	
Cabeza	46 ^†^	2,2	18 ^‡^	0,9	2	0,6	
Cuello	38 ^†^	1,8	5 ^‡^	0,3	1	0,3	
No realiza el procedimiento en niños	366	17,3	363	19,1	73	24,0	**0,000** ^§^
Sitio del cateterismo venoso en adultos *							
Mano	631 ^‡^	25,3	685 ^†^	27,7	113	26,9	
Brazo	687 ^‡^	27,5	777 ^†^	31,4	140 ^†^	33,3	
Antebrazo	941	37,7	916	37,0	154	36,7	
Pie	23	0,9	39	1,6	5	1,2	
Tobillo	5	0,2	21 ^†^	0,8	1	0,2	
Cabeza	4	0,16	1	0,04	1	0,2	
Cuello	145 ^†^	5,8	27 ^‡^	1,1	5 ^‡^	1,2	
No realiza el procedimiento en adultos	60 ^†^	2,4	9 ^‡^	0,4	1 ^‡^	0,2	**0,000** ^§^

* Pregunta que permitía múltiples respuestas

† Residual ajustado > 1,96

‡ Residual ajustado < -1,96

§ Prueba de razón de verosimilitud

|| Prueba Chi-cuadrado de Pearson para k muestras independientes

 Los DAV más citados fueron los que tenían dispositivo de seguridad. Respecto al material de estos dispositivos, los enfermeros mencionaron poliuretano y Teflón ^®^ , y en el grupo de técnicos y auxiliares de enfermería, más de la mitad no supo informar la constitución de los dispositivos utilizados en sus prácticas ( Tabla 4 ). 

 La mayoría de los profesionales utiliza el dispositivo 24 Gauge (G) en niños y el dispositivo 20G en adultos. También se puede observar que se adopta más de un criterio para la selección del dispositivo, siendo los más enumerados: calibre del vaso, fragilidad de la piel, duración del uso de la terapia y tipo de infusión ( Tabla 4 ). 

 En cuanto a los materiales para cubrir y estabilizar el DAV, la cinta adhesiva tipo Micropore ^®^ fue frecuentemente mencionada por los profesionales de Enfermería, seguida de la película transparente. En cuanto a la identificación del CIP, menos de la mitad de los profesionales indica el registro del horario, calibre y nombre del profesional responsable del procedimiento ( Tabla 4 ). 

 Hubo diferencias en el grupo de enfermeros y técnicos de enfermería que utilizaban todo tipo de materiales del dispositivo, DAV con calibre 22G en adultos y cobertura transparente. Entre los auxiliares, la diferencia se observó en las categorías Teflón ^®^ y poliuretano, grupo que desconocía el número del dispositivo y que utilizaba IV-Fix ^®^ ( Tabla 4 ). 

**Tabla 3:** - Técnica y procedimientos adoptados para la inserción de dispositivo vascular periférico por profesionales de Enfermería que realizan cateterismo intravenoso periférico (n = 2.584). Brasil, 2022

**Variable**	**Enfermero** Federal de São Paulo, Escola Paulista de Enfermagem **(n= 1.214)**		**Técnico de enfermería** (n= 1.166)		**Auxiliar de enfermería** **(n= 204)**		**p-valor**
	**N**	**%**	**n**	**%**	**n**	**%**	
**Técnica para torniquete del miembro** *							
Torniquete de uso único	273 ^†^	17,6	355 ^‡^	24,8	68 ^‡^	26,4	
Torniquete universal	675 ^‡^	43,4	594 ^†^	41,6	118	45,9	
Guante o parte del guante para procedimiento	514 ^‡^	33,1	426 ^†^	29,8	69	26,8	
Manos del profesional	92 ^‡^	5,9	53 ^†^	3,7	2 ^†^	0,8	**0,000** ^§^
**Tiempo de torniquete**							
30 seg	425	35,3	380	33,2	59	29,2	
1 minuto	397	33,0	403	35,2	78	38,6	
1 min 30 seg	69	5,7	91	7,9	11	5,4	
2 minutos	62	5,2	63	5,5	8	4,0	
No controla el tiempo	250	20,8	209	18,2	46	22,8	0,126 ^||^
**Distancia torniquete-área del cateterismo**							
Lo más cerca posible del sitio	582 ^‡^	48,5	502 ^†^	43,9	86	43,7	
Lo más distante posible del sitio	516	43,0	508		84	42,6	
Indiferente	103 ^†^	8,6	133 ^‡^	11,6	27	13,7	**0,028** ^||^
**Método de inserción del dispositivo**							
Directo	959 ^‡^	80,5	863 ^†^	76,1	143 ^†^	73,0	
Indirecto	200	16,8	218	19,2	38	19,4	
No sabe	33 ^†^	2,8	53	4,7	15 ^‡^	7,7	**0,002** ^||^
**Intentos máximos de cateterismo**							
1 intento	20 ^†^	1,7	49 ^‡^	4,3	11 ^‡^	5,5	
2 intentos	597 ^†^	50,0	620 ^‡^	54,2	113	56,8	
3 intentos	427	35,7	388	33,9	56 ^†^	28,1	
4 intentos	74 ^‡^	6,2	33 ^†^	2,9	7	3,5	
Tantos como sean necesarios	77 ^‡^	6,4	53 ^†^	4,6	12	6,0	**0,000** ^||^

* Pregunta que permitía múltiples respuestas

† Residual ajustado > 1,96

‡ Residual ajustado < -1,96

§ Prueba de razón de verosimilitud

|| Prueba Chi-cuadrado de Pearson para k muestras independientes

**Tabla 4:** - Dispositivos, cobertura/estabilización y registros de enfermería realizados por profesionales de Enfermería que realizan cateterismo intravenoso periférico (n = 2.584). Brasil, 2022

**Variable**	**Enfermero** **(n= 1.214)**		**Técnico de enfermería** (n= 1.166)		**Auxiliar de enfermería** **(n= 204)**		**p-valor** *
	**N**	**%**	**N**	**%**	**N**	**%**	
**Tipo de dispositivo** ^†^							
Simple	508	34,8	501	35,4	101	40,0	
Con dispositivo de seguridad	944	64,7	907	64,0	150	59,2	
No sabe	7	0,5	8	0,6	2	0,8	0,560
**Material del dispositivo** ^†^							
Teflón ^®^	393 ^‡^	28,4	231 ^§^	20,9	41 ^§^	21,6	
Poliuretano	421 ^‡^	30,5	149 ^§^	13,5	16 ^§^	8,4	
Vialon ^®^	62 ^‡^	4,5	30 ^§^	2,7	7	3,7	
No sabe	506	36,6	695	62,9	126	66,3	**0,000**
**Calibre del dispositivo en niño** ^†^							
18 Gauge	33	2,0	44	2,9	7	2,8	
20 Gauge	79	4,9	101	6,6	14	5,5	
22 Gauge	440	27,1	390	25,6	58	23,0	
24 Gauge	831	51,1	777	51,1	130	51,6	
No sabe	242	14,9	209	13,7	43	17,1	0,239
**Calibre del dispositivo en adulto** ^†^							
18 Gauge	316	15,6	314	15,7	50	14,4	
20 Gauge	839	41,3	774	38,6	131	37,9	
22 Gauge	637 ^§^	31,4	708 ^‡^	35,3	111	32,1	
24 Gauge	168	8,3	172	8,6	36	10,4	
No sabe	70 ^‡^	3,4	37 ^§^	1,8	18 ^‡^	5,2	**0,001**
**Criterio para seleccionar el dispositivo** ^†^							
Más grande disponible	29	0,9	25	0,9	3	0,6	
Menor disponible	64 ^‡^	1,9	38	1,3	2	0,4	
Calibre del vaso	996	30,4	899	31,6	151	31,4	
Fragilidad de la piel	704	21,5	617	21,7	108	22,5	
Tiempo de uso de la terapia	737	22,5	656	23,1	109	22,7	
Tipo de infusión	743	22,7	605	21,3	107	22,3	0,358
**Material utilizado para cobertura/estabilización** ^†^							
Cinta adhesiva tipo esparadrapo	318	14,9	298	15,1	52	15,6	
Cinta adhesiva tipo Micropore ^®^	616	28,9	600	30,5	119	35,6	
Película transparente	529 ^‡^	24,8	419 ^§^	21,3	75	22,4	
IV-Fix ^®^	279	13,1	270	13,7	28 ^§^	8,4	
Tegaderm ^®^	387	18,2	382	19,4	60	17,9	**0,026**
**Identificación del cateterismo intravenoso periférico** ^†^							
Fecha del procedimiento	94	7,9	88	8,2	19	9,3	
Horario del procedimiento	454	38,1	408	37,9	65	31,7	
Calibre del dispositivo	399	33,5	325	30,2	68	33,2	
Profesional responsable	243	20,4	254	23,6	53	25,8	0,214

* Prueba Chi-cuadrado de Pearson para k muestras independientes

† Pregunta que permitía múltiples respuestas

‡ Residual ajustado > 1,96

§ Residual ajustado < -1,96

## Discusión

En este estudio, hubo variaciones significativas en las prácticas relacionadas con el CIP implementadas por enfermeros, técnicos y auxiliares de enfermería que trabajan en las regiones geográficas del país, algunas de acuerdo con las recomendaciones actuales, mientras que otras no están en línea con las mejores prácticas para el CIP.

 Se observó que los enfermeros son los profesionales que menos asesoran a los familiares/tutores sobre el procedimiento de CIP. En este contexto, cabe señalar que la planificación del cuidado en TVI debe involucrar a la familia, ya que colabora en el proceso de tratamiento y recuperación de la salud de sus miembros ^(^ 12 ^)^ . Esta relación debe basarse en una asociación que respete los valores, preferencias, sentimientos y necesidades del paciente ^(^ 13 ^)^ . 

 En este sentido, proporcionar al familiar/tutor y/o paciente información sobre la correcta identificación de estos eventos ayuda a evitar el sufrimiento y malestar que provocan nuevos intentos de CIP, que pueden derivar en agotamiento del acceso venoso, miedo a las agujas y evitación hospitalaria ^(^ 14 ^)^ . 

 En términos de “preparación del paciente pediátrico antes del CIP”, en general, los profesionales de Enfermería de este estudio adoptan más de una estrategia, además de la atención prestada al manejo del dolor antes del cateterismo. Investigación transversal realizada en la Unidad de Cuidados Intensivos Neonatales de un hospital de Fortaleza-CE mostró cambios de comportamiento y fisiológicos en recién nacidos sometidos a CIP y que no utilizaron medidas no farmacológicas para aliviar el dolor ^(^ 15 ^)^ . 

 El cateterismo venoso es un procedimiento doloroso que conlleva una experiencia sensorial y emocional desagradable ^(^ 16 ^)^ . Por lo tanto, el equipo de Enfermería debe hacerlo menos estresante, a través de estrategias de control del dolor y aplicación de instrumentos para medir el dolor experimentado por el paciente ^(^ 17 ^)^ . 

En la presente investigación, el cuidado aséptico antes de cada intento de inserción del DAV contradice estándares de práctica mundialmente reconocidos, como la falta de higiene de las manos, la no utilización de nuevo material antisepsia o de un nuevo dispositivo. Respecto al cambio de guantes de procedimiento, se observa una mejora en los porcentajes, pero destaca que los profesionales pueden estar sustituyendo la higiene de manos por el uso de guantes. En el grupo de enfermeros esta tasa fue menor, pero aún representa un número notable de profesionales.

 La higiene de manos no puede sustituirse por el uso de guantes de procedimiento. Los profesionales justifican este cambio alegando que hay una pérdida de tacto al palpar la vena o realizar el CIP, y muchos utilizan el guante sólo al conectar el DAV al equipo o a la jeringa ^(^ 18 ^)^ . Varios estudios atestiguan que la adherencia profesional sigue siendo baja a pesar de tantas recomendaciones sobre la relevancia de las prácticas de higiene antes de la inserción del DAV. 

 Por lo tanto, observar la Teoría del Comportamiento Planificado (TCP) puede arrojar luz sobre esta y otras cuestiones relacionadas con el CIP. Se trata de un modelo predictivo de conducta en el que tres constructos psicológicos (actitud, norma subjetiva y percepción de control) explican que la intención de los profesionales es el antecedente inmediato del comportamiento ^(^ 19 ^)^ . 

 La actitud vincula el interés personal por realizar un determinado comportamiento con los resultados que se esperan de él. El profesional de Enfermería, a partir de sus creencias comportamentales, evalúa las consecuencias de no adoptar prácticas correctas de CIP, y el resultado de este análisis determinará su intención ^(^ 19 ^)^ . 

 La norma subjetiva se refiere a la percepción que tiene el profesional de la presión social para realizar una determinada conducta ^(^ 19 ^)^ . En muchos contextos laborales, la presión social sobre los enfermeros para que el equipo realice procedimientos asépticos antes de cada intento de CIP es inexistente, y ellos mismos no adoptan esa postura. 

 La percepción de control demuestra el grado en que el profesional se siente capaz de realizar un comportamiento y puede ser influenciada por actitudes y normas subjetivas. Incluso si el profesional de Enfermería está a favor de adoptar prácticas adecuadas de CIP, un individuo puede sucumbir a la presión social ejercida por otros colegas o a un bajo control ^(^ 19 ^)^ . 

 En cuanto a la evaluación de la red venosa, los profesionales refieren la utilización de más de un criterio. El método tradicional y la aplicación de calor fueron los más citados por los técnicos y auxiliares de enfermería, mientras que los enfermeros destacan el uso de tecnologías como la ecografía vascular y el Venoscopio ^®^ . 

 En el método tradicional, los profesionales se guían por puntos de referencia anatómica, inspección y palpación de la vena periférica. A pesar de ser comúnmente utilizado en la práctica clínica por profesionales de nivel secundario y técnico, un estudio de revisión sistemática encontró superioridad en el uso de la ecografía en relación al cateterismo tradicional ^(^ 20 ^)^ . Este dato resalta que la tecnología de visualización de venas permite mayor asertividad, agilidad y seguridad, menos intentos para un cateterismo exitoso, menos complicaciones, reducción del tiempo del procedimiento y mayor satisfacción del paciente ^(^ 21 ^)^ . 

 Un resultado preocupante es que algunos técnicos y auxiliares de enfermería afirman utilizar la ecografía vascular en sus prácticas profesionales, a pesar de que el uso de esta tecnología es exclusivo del enfermero. Además, aún prevalece la cultura de que el CIP es un procedimiento simple y puede ser realizado por cualquier miembro del equipo de Enfermería ^(^ 22 ^)^ . 

 Por lo tanto, es necesario que el enfermero asuma la responsabilidad legal de la inserción del dispositivo periférico, ya que asociadas a la complejidad del procedimiento existen condiciones que tornan la venopunción periférica difícil (VPD), como niños prematuros, desnutridos, obesos o con enfermedades crónicas y adultos con alta complejidad asistencial ^(^ 23 ^)^ . En este último caso, la prevalencia de VPD es del 59,3% y varía según la condición clínica del paciente ^(^ 24 ^)^ . 

La mayoría de los profesionales de Enfermería mencionó el uso de herramientas clínicas para evaluar la red venosa difícil. Aunque el porcentaje fue mayor en el grupo de auxiliares de enfermería, se observó que desconocían la definición de herramienta clínica, ya que enumeraron como sinónimos el uso de anatomía, tecnología, torniquete, dispositivo, entre otros.

 La adopción de instrumentos de medición en la práctica clínica, como escalas, protocolos con recomendaciones, *Bundles* y flujogramas para evaluar la dificultad del CIP puede contribuir a la calidad de la atención de Enfermería, con la consiguiente reducción de la tasa de fracaso y agotamiento de los vasos sanguíneos, ya que los cuidados vasculares son importantes para preservar la salud de los vasos a lo largo de la vida de un individuo ^(^ 25 ^)^ . 

Otros cuidados importantes incluyen la selección de dispositivos de calibre apropiado, la elección del sitio de las venas periféricas que apoyan la terapia a implementar, la técnica de inserción del DAV y la administración de soluciones y medicamentos en cantidades y concentraciones apropiadas.

 Los profesionales de este estudio destacaron varios criterios para la selección de venas periféricas. El tiempo de uso de la TVI periférica y el calibre del dispositivo fueron citados por todas las categorías profesionales. Los enfermeros también enumeraron la clasificación de la medicación, mientras que los técnicos y auxiliares de enfermería agregaron la palpabilidad de la vena. Durante el tratamiento de pacientes hospitalizados también se debe considerar el tipo de solución a infundir, el tiempo de infusión y el estado de las venas ^(^ 26 ^)^ . 

Además, el equipo de Enfermería debe monitorear los efectos y conocer la naturaleza de los medicamentos, para evitar complicaciones locales y/o sistémicas. En el presente estudio, ese criterio fue poco considerado por los profesionales de Enfermería.

Otro aspecto relevante pocas veces mencionado por el equipo de Enfermería es el de la preferencia del paciente. Quizás la comunicación verbal entre profesional y paciente antes de la inserción del DAV no sea rutinaria dentro del servicio de salud.

 En el presente estudio, los profesionales de Enfermería mencionaron manos, brazo y antebrazo como los principales sitios de elección para el cateterismo venoso en niños. Estudio descriptivo desarrollado en Unidades de Cuidados Intensivos Neonatales (UCIN) y Unidades de Cuidados Intensivos Pediátricos (UCIP) mostró que el 55,5% de los profesionales de Enfermería en la UCIN y el 34,6% en la UCIP eligieron las venas del dorso de las manos como primera opción para el CIP ^(^ 27 ^)^ . 

 Estos datos concuerdan con la *Infusion Nurses Society* (INS) estadounidense, que recomienda elegir los vasos con mayor probabilidad de durar toda la terapia prescrita, como los miembros superiores y el dorso de las manos de los pacientes pediátricos, evitando las zonas de flexión para no limitar el movimiento del niño ^(^ 7 ^)^ . 

 Por el contrario, la INS Brasil afirma que el primer intento de CIP debe iniciarse en la región más distal, considerando las particularidades de cada fármaco y las posibles complicaciones ^(^ 28 ^)^ . También recomienda la elección de las venas de la cabeza en niños menores de tres años y, si no caminan, el uso de las venas del pie ^(^ 7 ^)^ . 

 Una investigación transversal global realizada en 278 hospitales de 47 países con datos de 4206 niños identificó la mano (51%; n= 2143) como el sitio más accedido para la colocación del DAV. Sin embargo, en América del Norte, Australia y Nueva Zelanda, la fosa antecubital (tasa variando entre 21,4 y 24,5%) fue la región más mencionada ^(^ 29 ^)^ . 

 En urgencias se accede con frecuencia a esta región debido a la posibilidad de infusión rápida de grandes volúmenes y, por ello, algunos autores recomiendan este sitio por el mayor calibre de las venas ^(^ 12 ^)^ . En cambio, otros no lo recomiendan, debido a la limitación de movilidad de la extremidad del paciente, a menos que se utilicen dispositivos venosos flexibles o este sea el único acceso venoso disponible ^(^ 26 ^)^ . 

 En los adultos, las venas cefálica y basílica del antebrazo son los sitios preferidos para el CIP. La mayoría de los profesionales entrevistados mencionó el brazo y el antebrazo, pero algunos eligieron los miembros inferiores como sitio de cateterismo. Este hallazgo, a pesar del bajo porcentaje, causa preocupación, debido al mayor riesgo de embolia, tromboflebitis e infección por CIP en las venas de la pierna/pie en adultos ^(^ 26 ^)^ . 

Después de la selección de la vena, se adoptan técnicas y procedimientos para facilitar la inserción del DAV, como el uso de un torniquete, que permite la venodilatación y facilita la visualización y el proceso de CIP. Respecto al tiempo que permanece el torniquete en el paciente, casi todos los enfermeros informaron que lo dejan puesto por 30 segundos, mientras que los técnicos y auxiliares de enfermería lo mantienen por hasta 1 minuto. Sin embargo, un porcentaje importante de profesionales no controla su tiempo.

 El no control del tiempo puede provocar errores de diagnóstico como hemólisis, aumento de los niveles de potasio y/o calcio, además de generar complicaciones durante el cateterismo, como hematomas, hormigueos y, en casos extremos, el signo de Trousseau ^(^ 30 ^)^ . 

 También hay casos de torniquetes olvidados en las extremidades de los pacientes después de intentar colocar el DAV. Por lo tanto, el enfermero debe estar alerta ante signos de retención del torniquete, que incluyen dolor en las extremidades, hormigueo, edema, infusión intravenosa que fluye mal, fuga en el sitio de inserción del DAV y/o en los sitios de cateterismo ^(^ 31 ^)^ . 

 Otro factor predisponente para la aparición de complicaciones durante la TVI es la técnica de inserción del DAV, ya sea directa o indirecta. Los profesionales de este estudio mencionaron el uso del método directo, pero un pequeño porcentaje de auxiliares y técnicos de enfermería no supo identificar el método de inserción del dispositivo. La evidencia indica que el método directo está más asociado con la aparición de complicaciones intravenosas ^(^ 32 ^)^ . 

Ante esto, los servicios de salud han buscado garantizar un cuidado libre de riesgos y daños. Y en este contexto, los enfermeros desempeñan un papel clave en la evaluación del riesgo de acceso venoso periférico difícil, para evitar numerosos intentos fallidos de cateterismo.

 Un estudio realizado en Portugal demostró que los enfermeros necesitan de dos a ocho intentos de cateterismo para insertar con éxito un DAV, con tasas que varían entre el 19,4% y el 23,7% ^(^ 33 ^)^ . Este número tiende a aumentar a un promedio de cinco intentos por persona, pudiendo variar entre uno y 20 intentos si se considera todo el período de tratamiento del paciente hospitalizado ^(^ 33 ^)^ . 

 La *Agência Nacional de Vigilância Sanitária* (ANVISA) recomienda hasta un máximo de dos intentos por profesional con la ayuda de tecnologías de visualización de venas, y limita el número de intentos a un máximo de cuatro punciones (dos por profesionales diferentes) ^(^ 25 ^)^ . 

 Los intentos repetidos de CIP afectan negativamente la experiencia general del paciente y pueden causar daño al vaso, lo que aumenta la posibilidad de utilizar dispositivos de acceso venoso central ^(^ 3 ^)^ . Los profesionales participantes de esta investigación mencionaron haber realizado tres intentos, lo que ya contradice la normativa vigente, siendo esta tasa mayor en el grupo de enfermeros. 

 En cuanto a las prácticas relacionadas al cuidado y cobertura/estabilización del dispositivo, se observó que los materiales del dispositivo más utilizados por los enfermeros fueron poliuretano y Teflón ^®^ . Algunos de los técnicos y auxiliares de enfermería utilizaban dispositivo de Teflón ^®^ , y más de la mitad no supo informar el tipo de material (un porcentaje considerable de enfermeros también entran en esta categoría). 

 El desconocimiento sobre la constitución del DAV aumenta el riesgo de complicaciones, ya que la evidencia muestra que los dispositivos fabricados con poliuretano se asocian con bajas incidencias de complicaciones infecciosas en comparación con los fabricados con policloruro de vinilo y polietileno ^(^ 34 ^)^ . 

En general, los profesionales de Enfermería utilizan dispositivos de 24G para niños y de 20G para adultos. Se observa que la elección del calibre en la población pediátrica resultó en un porcentaje considerable de profesionales que desconocían el número del dispositivo, especialmente auxiliares de enfermería y enfermeros.

 Para los pacientes pediátricos, se recomienda el uso de calibres 22G y 24G, pero un pequeño porcentaje de los encuestados utiliza calibres 18G y 20G. Los dispositivos de menor calibre se asocian con menos complicaciones, además de provocar menos agresión mecánica a la pared de la vena por parte de la cánula y menor obstrucción del flujo sanguíneo dentro del vaso ^(^ 9 ^)^ , pero esta afirmación debe tener en cuenta la edad del paciente y las características de la red venosa. 

Las venas de los niños son más frágiles en comparación con las de los adultos, por lo que evaluar los riesgos y beneficios de cada tipo de dispositivo, como calibre, constitución y criterios de selección, es fundamental. En este último ítem, los profesionales de Enfermería, en su mayoría, eligieron el tiempo de utilización de la terapia como criterio principal para la selección del dispositivo.

 Luego del CIP, los datos destacaron que la mayoría de los profesionales de Enfermería, incluidos los enfermeros, utiliza cinta adhesiva tipo Micropore ^®^ para estabilizar y cubrir el dispositivo. Este hallazgo es preocupante, dado que el uso de cintas adhesivas no estériles es una práctica ampliamente observada en los países en desarrollo. 

 Es habitual ver a numerosos profesionales de enfermería que cortan tiras de esparadrapo antes de realizar el CIP y las fijan en su propio uniforme, en la bandeja o en la mesa auxiliar del paciente. El propósito de la cobertura es proteger el sitio de inserción del DAV y reducir el riesgo de infección, por lo que esta práctica de rutina contamina la cinta después de abrir el embalaje original. La cinta adhesiva debe ser estéril y cambiarse siempre que esté húmeda, sucia, suelta o su integridad se vea comprometida. Si se sospecha contaminación, el cambio debe ser inmediato ^(^ 7 ^)^ . 

Otro aspecto significativo después de la inserción del DAV es su identificación, que permite la comunicación entre el equipo de Enfermería y posibilita la continuidad de los cuidados. En el presente estudio, se observa que el equipo no se preocupa por registrar la fecha del procedimiento, ya que los porcentajes de adopción de esa práctica fueron muy bajos, incluso en el grupo de enfermeros. La mayoría de los profesionales registran en el dispositivo la hora del procedimiento, el calibre del dispositivo y el nombre del profesional responsable.

 No registrar la fecha puede contribuir a la alta prevalencia de catéteres inactivos, definidos como no utilizados en las 24 horas anteriores y no previstos para su uso en las próximas 24 horas. La literatura indica que alrededor del 14 al 50% de los periféricos se mantienen con el paciente “por si acaso” son necesarios ^(^ 35 ^)^ . 

 En algunas instituciones, la duración de estancia recomendada para el DAV es de 96 horas. Para ANVISA, el cambio no debe ser inferior a 96 horas, y la evaluación rutinaria del equipo de Enfermería permitirá tomar la decisión de conservar el dispositivo por más tiempo, o cuando esté clínicamente indicado ^(^ 9 ^)^ . 

Además, la Organización Mundial de la Salud (OMS) agrega que el registro del CIP es un requisito de identificación del paciente y un objetivo que garantiza la seguridad en los servicios de salud y mitiga la ocurrencia de errores.

La datación del cateterismo permite el seguimiento del DAV desde su inserción, además de posibilitar comprobar su validez en situaciones en las que está prevista su retirada. Y, en caso de complicación, la posibilidad de evaluar los motivos y tomar medidas para evitar que empeore.

Los resultados de este estudio difunden mejores prácticas relacionadas con la inserción del DAV periférico, particularmente en lo que respecta al cuidado antes de cada intento de inserción, que involucra preparación del paciente, evaluación de la red venosa, técnicas y procedimientos adoptados, dispositivos, cobertura/estabilización y registros de enfermería.

Como limitación del estudio, se destaca la elección del diseño epidemiológico que analizó las prácticas de manera específica, lo que tiene poco poder para generar evidencia robusta sobre el CIP realizado por los profesionales de Enfermería. Además, el muestreo no probabilístico interfiere con la validez externa del estudio.

## Conclusión

La mayoría de los profesionales de Enfermería no involucra al paciente y a su familia en el cuidado, adopta estrategias para preparar al paciente antes del cateterismo y no mantiene cuidados asépticos. Al evaluar la red venosa, el porcentaje de profesionales que no utilizan métodos de evaluación y/o herramientas clínicas fue mucho menor.

Muchos profesionales no controlan el tiempo adecuado del torniquete y tienen dificultades para identificar la distancia correcta entre el torniquete y la zona de cateterismo. Además, el método de inserción del dispositivo más adoptado es precisamente el que más se asocia con la aparición de complicaciones intravenosas, y casi la mitad de los enfermeros y técnicos de enfermería intentan punzar al mismo paciente tres o más veces.

 En cuanto a las características del dispositivo, los profesionales tenían conocimientos similares sobre el tipo de dispositivo y calibre utilizado en niños y adultos. Existe una gran cantidad de técnicos y auxiliares de enfermería que desconoce la constitución de los dispositivos utilizados en el ambiente laboral. La mayoría de los profesionales utilizaba cinta adhesiva tipo Micropore ^®^ para cubrir y estabilizar el DAV, y casi la totalidad de las tres categorías profesionales investigadas no registraba la fecha del CIP. 

A la vista de los resultados encontrados, se desprende que los técnicos y auxiliares de enfermería son los profesionales que menos cumplen con lo recomendado en guías reconocidas. Sin embargo, la práctica de los enfermeros también presenta desviaciones en relación a la evidencia científica, y su desempeño no fue muy diferente al de los profesionales de nivel secundario/técnico. Así, se revelaron debilidades en el cuidado ofrecido al paciente antes de la inserción del dispositivo de acceso vascular periférico que podrían comprometer la seguridad y causar complicaciones.

Se entiende que es necesario implementar acciones educativas y de formación teórico-práctica para el equipo de Enfermería, incluidos los enfermeros. Por lo tanto, el conocimiento técnico-científico puede garantizar la efectividad en el tratamiento y la calidad del cuidado brindado.

# Translation: Prática nacional dos profissionais de Enfermagem na inserção de dispositivos de acesso vascular periférico *

**DOI:** 10.1590/1518-8345.6673.4315

**Published:** 2024-09-23

**Authors:** Bianka Sousa Martins Silva, Luciano Marques dos Santos, Patrícia Kuerten Rocha, Aline Nair Biaggio Mota, Ariane Ferreira Machado Avelar, Denise Miyuki Kusahara

**Affiliations:** 1 Universidade Federal de São Paulo, Escola Paulista de Enfermagem, São Paulo, SP, Brasil; 2 Universidade Estadual de Feira de Santana, Departamento de Saúde, Feira de Santana, BA, Brasil.; 3 Universidade Federal de Santa Catarina, Departamento de Saúde, Santa Catarina, SC, Brasil.; 4 BIOTRONIK Comercial Médica, Medical Affairs, São Paulo, SP, Brasil.

**Keywords:** Cateterismo Periférico, Infusões Intravenosas, Dispositivos de Acesso Vascular, Equipe de Enfermagem, Cuidado de Enfermagem, Competência Profissional

## Abstract

**Objetivo:**

identificar e comparar a prática dos profissionais de Enfermagem quanto à inserção de dispositivos de acesso vascular periféricos, de acordo com a categoria profissional.

**Método:**

estudo seccional descritivo realizado no período entre julho de 2021 e maio de 2022 com 2.584 profissionais de Enfermagem, por meio de questionário validado por três juízes com expertise em terapia intravenosa, contendo variáveis relacionadas à cateterização e ao dispositivo de acesso vascular. Foi realizada análise descritiva e inferencial.

**Resultados:**

a maioria dos profissionais não prepara o paciente nem realiza alguns cuidados essenciais antes da tentativa de cateterização intravenosa periférica. Quanto ao local preferencial de cateterização, destacam-se as mãos, o braço e o antebraço. Não há controle do tempo de garroteamento e o paciente é puncionado mais de três vezes. Os materiais dos dispositivos mais utilizados são poliuretano e Teflon ^®^ , é adotado mais de um critério para a seleção do dispositivo, e a fita adesiva do tipo Micropore ^®^ foi a cobertura mais citada pelos profissionais de Enfermagem. A identificação da cateterização não foi adequada.

**Conclusão:**

técnicos e auxiliares de enfermagem são os profissionais que menos atendem ao que está preconizado nas diretrizes reconhecidas. A prática dos enfermeiros também apresenta desvios em relação às evidências científicas.

## Destaques

**(1)** A maioria dos profissionais de Enfermagem não envolve o paciente e a família no cuidado.

**(2)** Técnicos/auxiliares são os que menos atendem às normas preconizadas.

**(3)** Enfermeiros também apresentam desvios em relação às evidências científicas.

**(4)** A atuação do enfermeiro não foi muito diferente dos profissionais de nível médio.

**(5)** Há fragilidades no cuidado oferecido ao paciente.

## Introdução

 A cateterização intravenosa periférica (CIP) é amplamente realizada em pacientes nos mais diversos ambientes de atendimento à saúde, principalmente para a inserção de dispositivos de acesso vascular (DAV). Este procedimento possibilita a implementação de terapêuticas diversas, como a administração de fluidos, medicamentos, hemocomponentes e nutrição diretamente no sistema circulatório ^(^ 1 ^)^ . 

 Pesquisa de corte transversal multinacional realizada em cinco países da América Latina indica que cerca de 70% dos pacientes hospitalizados são submetidos a CIP ^(^ 2 ^)^ . Apesar de rotineira, esta prática pode ocasionar complicações que resultam em morbimortalidade, aumento do tempo de hospitalização e custos significativos que impactam o estado clínico e a evolução do paciente ^(^ 3 ^)^ . A (re)inserção do DAV é um procedimento estressante para o paciente e a família durante a hospitalização, e está associado ao aumento da fobia de agulhas e resistência de adultos em buscar atendimento nos serviços de saúde ^(^ 4 ^-^ 5 ^)^ . 

 A complexidade da CIP e sua alta taxa de complicações associadas indicam que o enfermeiro deveria ser o protagonista e atuar em todas as etapas do processo, desde a seleção do tipo, a instalação, até a remoção do DAV, em consonância com as recomendações e diretrizes internacionais reconhecidas para a prática de Enfermagem ^(^ 6 ^-^ 7 ^)^ . 

 No ambiente laboral, todos os profissionais de Enfermagem insertores do DAV periférico devem ser treinados de forma eficaz, de modo a fornecer aos pacientes um cuidado de alta qualidade guiado pelas melhores evidências ^(^ 8 ^)^ . 

 Conhecer as práticas de inserção de DAV periféricos executadas por profissionais de Enfermagem é imperativo para a garantia da eficácia do tratamento e do cuidado prestado, além de evitar o aparecimento de complicações relacionadas à terapia intravenosa (TIV). Evidências apontam que no período de junho de 2021 a maio de 2022 foram notificados cerca de 39.994 eventos adversos/complicações envolvendo dispositivos de acesso vascular nos serviços de saúde ^(^ 9 ^)^ . 

Assim, considerando a importância da CIP e a necessidade de avaliar a execução deste procedimento, no intuito de melhorar a qualidade da assistência, surge como objetivo deste estudo identificar e comparar a prática dos profissionais de Enfermagem quanto à inserção de dispositivos de acesso vascular periférico, de acordo com a categoria profissional.

## Método

### Delineamento do estudo

 Trata-se de um estudo seccional do tipo *survey* descritivo. Para descrição e relato do estudo foi utilizado como referência o *Strengthening the Reporting of Observational Studies in Epidemiology* (STROBE). 

### Local

Realizado nas cinco macrorregiões do Brasil.

### Período

A coleta de dados ocorreu no período entre julho de 2021 e maio de 2022.

### População e critério de seleção

 A população do estudo foram enfermeiros, técnicos e auxiliares de enfermagem residentes nas cinco macrorregiões do Brasil. Dados do Conselho Federal de Enfermagem (Cofen) (2022) registravam um total de 2.513.428 profissionais de Enfermagem, sendo 613.827 (24,42%) enfermeiros, 1.463.072 (58,21%) técnicos de enfermagem e 436.529 (17,37%) auxiliares de enfermagem ^(^ 10 ^)^ . 

Foram incluídos os profissionais que atuavam no cuidado direto ao paciente submetido à CIP e que realizavam este procedimento durante sua rotina de atividade. Profissionais com até um ano após a conclusão da graduação ou curso técnico e sem experiência prévia em CIP não foram incluídos, e excluíram-se aqueles que não informaram sua categoria profissional no questionário.

### Amostra

 Para o cálculo amostral, foi considerada uma população de 2.513.428 profissionais de Enfermagem, precisão de 3% e frequência de 80% de CIP. A amostra mínima foi estimada em 837 participantes. O cálculo do tamanho amostral foi realizado na ferramenta *OpenEpi* , disponibilizada gratuitamente na internet ( https://www.openepi.com/Menu/OE_Menu.htm ). 

 Inicialmente, foi enviada uma mensagem de *e-mail* para o Cofen solicitando acesso às informações dos enfermeiros pediatras do Brasil. O pedido gerou o Processo Administrativo COFEN nº 0940/2017, que foi encaminhado ao Setor de Procuradoria Geral. Este emitiu o parecer jurídico nº 10/2018, favorável ao fornecimento dos dados após formalização de Termo de Compromisso entre o Cofen e a Universidade Federal de São Paulo/Programa de Pós-Graduação em Enfermagem. 

 O Cofen disponibilizou um banco de dados contendo 613.987 inscrições ativas de enfermeiros e técnicos de enfermagem. As únicas informações presentes eram a “categoria profissional” e o “endereço eletrônico ( *e-mail* )”, o que dificultou a identificação dos enfermeiros pediatras. Por esta razão, o público foi ampliado para crianças e adultos. 

 Além disso, havia, também, muitos dados duplicados e inconsistências, como a repetição de um mesmo *e-mail* cerca de 16.644 vezes (2.71%). Desta forma, foi utilizada amostra não probabilística intencional, que incluiu uma fase de cadeia de referência ou bola de neve ^(^ 11 ^)^ . Esta técnica foi utilizada de forma a complementar os dados do Cofen, que não representavam a população de Enfermagem. 

 O primeiro passo no método de amostragem em bola de neve foi encontrar indivíduos pertencentes à população-alvo do estudo, por meio das redes sociais ( *Instagram* ^®^ , *Facebook* ^®^ ) e *WhatsApp* ^®^ , grupos de pesquisa e profissionais de contato dos pesquisadores. 

 Esses profissionais foram denominados de semente da amostra, e deram origem a outros profissionais amostrados. A partir da semente, começou o processo da bola de neve, no qual os primeiros profissionais foram considerados a onda zero. Os profissionais indicados pela onda zero que faziam parte da população alvo e que não faziam parte da onda zero constituíram a onda um. Foi solicitado que estes indivíduos repassassem o *link* da pesquisa para outros profissionais, e assim sucessivamente. 

### Variáveis do estudo

Foram investigadas as variáveis relacionadas ao profissional (categoria profissional, tempo de formação e número de DAV periférico inserido em um turno de 12 horas de trabalho), variáveis relativas à CIP (explica o procedimento ao familiar/responsável e paciente, estratégia e preparação do paciente, recurso para manejo da dor antes da cateterização), variáveis relativas às tentativas de CIP (higieniza as mãos, troca as luvas a cada tentativa, utiliza novo material para antissepsia a cada tentativa de cateterização, troca o dispositivo a cada tentativa de CIP), variáveis relativas à inserção do DAV (local da cateterização venosa em crianças e adultos, método de inserção do dispositivo, método para avaliação da rede venosa, uso de ferramenta clínica para avaliação de rede venosa difícil, critérios para a seleção de veias periféricas, número de tentativas de cateterização, garroteamento do membro, tempo de garroteamento e distância do torniquete da área de cateterização), variáveis relacionadas ao dispositivo e cobertura (tipo e material do dispositivo, calibre do dispositivo usado em criança e adulto, critério para seleção do dispositivo e material usado para cobertura/estabilização do dispositivo) e variáveis relacionadas ao registro (identificação da CIP).

### Coleta dos dados e instrumentos

 A coleta de dados foi realizada por meio de questionário contendo questões sobre o preparo do paciente, cuidados realizados por profissionais de Enfermagem antes da CIP, método de avaliação e seleção de veias periféricas, técnica e procedimentos adotados para inserção do DAV, dispositivos, cobertura/estabilização e registros de enfermagem. O questionário foi elaborado pelos pesquisadores com base nas recomendações dos *Infusion Therapy Standards of Practice* ^(^ 7 ^)^ e avaliado por três juízes com expertise em terapia intravenosa. A confiabilidade do instrumento foi verificada através do alfa de Cronbach, com valor igual a 0,82, demonstrando uma consistência interna quase perfeita. 

 Os questionários foram enviados aos participantes por *e-mail* , mala direta ( *Zievo* ^®^ ), redes sociais ( *Instagram* ^®^ e/ou *Facebook* ^®^ ), *WhatsApp* ^®^ e *sites* dos Conselhos Regionais de Enfermagem (COREN) de algumas regiões do Brasil. O envio foi gerenciado através da ferramenta *Research Electronic Data Capture* ( *REDCap* ^®^ ), disponibilizada por uma instituição federal de ensino do estado de São Paulo. 

### Análise dos dados

Realizou-se a análise descritiva dos dados, sendo as variáveis categóricas descritas por meio de frequências absolutas e relativas. Para comparar as frequências das variáveis qualitativas dos três grupos foi utilizado o teste Qui-quadrado para k amostras independentes, considerando nível de significância de 5% e intervalo de confiança (IC) 95%. Para variáveis com frequência esperada < 20% foi utilizado o teste da razão de verossimilhanças.

Foi aplicada a análise de resíduos ajustados (> 1,96 ou < -1,96) para identificação das categorias que apresentaram maior diferença entre as contagens esperadas e contagens reais em relação ao tamanho amostral. Resíduos positivos indicavam frequência observada maior que a esperada e resíduos negativos, o inverso.

 O valor de p foi calculado no *OpenEpi* versão 3.0, utilizando-se a opção cálculos Tabela L x C, e a análise de dados foi executada no *Statistical Package for the Social Sciences* (SPSS), versão 26.0. 

### Aspectos éticos

 A pesquisa atendeu aos pressupostos da Resolução 466/12 e foi aprovada pelo Comitê de Ética em Pesquisa (CEP) da Universidade Federal de São Paulo sob CAAE n.º 79646317.7.0000.5505 e parecer n.º 3.274.729. O termo de consentimento livre e esclarecido contendo informações sobre a pesquisa foi enviado pelo *REDCap* ^®^ . 

## Resultados

Participaram 1.214 (41%) enfermeiros, 1.166 (39,4%) técnicos e 204 (6,9%) auxiliares de enfermagem, totalizando 2.960 participantes. Deste quantitativo, 376 (12,7%) foram excluídos por não declararem a categoria profissional, resultando em amostra final de 2.584 participantes. A maioria eram mulheres residentes da macrorregião Sudeste. A idade média dos enfermeiros foi de 39,52 (± 8,74) anos, dos técnicos 39,66 (± 9,22) anos, e dos auxiliares de enfermagem 40,61 (± 10,57) anos.

 Na Tabela 1 , observa-se que grande parte dos profissionais não explica o procedimento de CIP para o familiar e/ou responsável, nem para os pacientes adultos, mas utilizam diversas estratégias de preparação do paciente pediátrico antes da CIP, bem como recursos para o manejo da dor. 

 Quase a totalidade dos respondentes não higieniza as mãos antes da inserção do DAV, e um pouco mais da metade dos enfermeiros e auxiliares de enfermagem trocam as luvas de procedimento antes da tentativa de CIP, sendo que no grupo dos técnicos de enfermagem esta distribuição foi mais equitativa. Além disso, um número considerável de profissionais de Enfermagem não utiliza novo material para antissepsia a cada tentativa de CIP, nem um novo dispositivo ( Tabela 1 ). 

**Tabela 1:** - Preparo do paciente e cuidados realizados por profissionais de Enfermagem antes de cada tentativa de inserção do dispositivo de acesso vascular segundo categoria profissional (n = 2.584). Brasil, 2022

**Variável**	**Enfermeiro** **(n= 1.214)**		**Técnico de enfermagem (n= 1.166)**		**Auxiliar de enfermagem (n= 204)**		**p-valor**
	**n**	**%**	**n**	**%**	**n**	**%**	
**Explicação do procedimento para o familiar/responsável**							
Sim	323	26,6*	438	37,6 ^†^	75	36,8	
Não	891	73,4 ^†^	728	62,4*	129	63,2	**0,000** ^‡^
**Explicação do procedimento para o paciente**							
Sim	92	7,6	73	6,3	7	3,4*	
Não	112	92,4	1093	93,7	197	96,6	0,068 ^‡^
**Estratégia de preparo da criança** ^§^							
Brinquedo terapêutico	186	17,9 ^†^	115	20,9*	17	20,7	
Livretos	45	4,3 ^†^	22	4,0*	5	6,1	
Realidade virtual	42	4,0	30	5,4	4	4,9	
Amamentação	131	12,6*	54	9,8 ^†^	7	8,5 ^†^	
Contato pele a pele	185	17,8 ^†^	116	21,1*	15	18,3*	
Enrolamento	201	19,3 ^†^	83	15,1*	13	15,8*	
Solução adocicada	148	14,2 ^†^	45	8,2*	4	4,9*	
Não utiliza	102	9,8	85	15,4	17	20,7	**0,000** ^||^
**Uso de recursos para manejo da dor antes da cateterização**							
Sim	749	62,7	657	58,3*	134	67,0	
Não	446	37,3	470	41,7	66	33,0	**0,019** ^‡^
**Higienização das mãos**							
Sim	7	0,6	2	0,2	-	-	
Não	119	99,4	1144	99,8	201	100	0,123 ^||^
**Troca de luvas a cada tentativa de CIP** ^¶^							
Sim	775	64,4 ^†^	571	49,9	112	56,0	
Não	429	35,6	573	50,1 ^†^	88	44,0	**0,000** ^‡^
**Utilização de novo material para antissepsia**							
Sim	133	11,1 ^†^	75	6,5	8	4,0	
Não	107	88,9	1071	93,5 ^†^	192	96,0 ^†^	**0,000** ^‡^
**Utilização de novo dispositivo**							
Sim	114	9,5 ^†^	66	5,8*	18	9,0	
Não	108	90,5*	1078	94,2 ^†^	183	91,0	**0,003** ^‡^

* Resíduo ajustado < -1,96

† Resíduo ajustado > 1,96

‡ Teste Qui-quadrado de Pearson para k amostras independentes

§ Questão que admitiu respostas múltiplas

|| Teste da razão de verossimilhança

¶ CIP = Cateterização intravenosa periférica

 Os critérios para a seleção de veias periféricas mais citados foram o tempo de uso da terapia intravenosa periférica e o calibre do dispositivo. Quanto ao local preferencial de cateterização venosa em crianças e adultos, os profissionais de Enfermagem destacaram as mãos, o braço e o antebraço ( Tabela 2 ). 

 Grande parte dos enfermeiros e técnicos avalia a rede venosa por meio da aplicação de calor, enquanto auxiliares de enfermagem utilizam métodos tradicionais, como palpação e visualização do vaso. A maioria dos profissionais de Enfermagem utiliza ferramentas clínicas para a avaliação de rede venosa difícil ( Tabela 2 ). 

 Houve diferenças estatisticamente significativas entre os profissionais que avaliavam a rede venosa utilizando venoscópio, ultrassonografia vascular ou aplicação de calor, ferramentas para avaliação da rede venosa difícil, e que listaram o tempo de uso da TIV, a classificação e natureza do medicamento e a visibilidade da veia como critérios para a seleção de veias periféricas. Na variável local de cateterização em crianças e adultos, também se observam diferenças consideráveis entre os três grupos, conforme os valores dos resíduos ajustados apresentados na Tabela 2 . 

 A técnica de garroteamento mais citada foi o torniquete universal com o tempo variando entre 30 segundos e 1 minuto. Com relação à distância entre o torniquete e a área de cateterização, houve distribuição equitativa. Nota-se, também, um número expressivo de profissionais de Enfermagem que utiliza o método direto de inserção do dispositivo e adotam duas ou três tentativas de cateterização ( Tabela 3 ). 

**Tabela 2:** - Avaliação da rede venosa e seleção de veias periféricas segundo categoria profissional (n = 2.584). Brasil, 2022

**Variável**	**Enfermeiro** **(n= 1.214)**		**Técnico de enfermagem (n= 1.166)**		**Auxiliar de enfermagem (n= 204)**		**p-valor**
	**n**	**%**	**n**	**%**	**n**	**%**	
**Método para avaliação da rede venos** a*							
Método tradicional	54	11,5	69	21,9	12	30,0	
Venoscópio ^®^	67 ^†^	14,2	42 ^†^	13,3	1 ^†^	2,5	
*Accuvein* ^®^	5	1,1	7	2,2	1	2,5	
*Vein Viewer* ^®^	45	9,6	29	9,2	2	5,0	
Ultrassom vascular	71 ^†^	15,6	22 ^‡^	7,0	2 ^‡^	5,0	
Aplicação de calor	150 ^†^	31,9	81 ^‡^	25,7	11 ^‡^	27,5	
Torniquete duplo	46	9,8	30	9,5	3	7,5	
Torniquete triplo	7	1,8	3	0,95	1	2,5	
Não utiliza	25	5,3	32	10,2	7	17,5	**0,000** ^§^
**Utilização de ferramenta clínica para avaliação de rede venosa difícil**							
Sim	1069 ^†^	88,8	964 ^‡^	84,5	188 ^†^	92,5	
Não	135	11,2	177	15,5	15	7,4	**0,000** ^||^
**Critérios para seleção de veias periféricas** *							
Tempo de uso da terapia intravenosa periférica	921 ^†^	19,9	700 ^‡^	18,8	116 ^‡^	19,2	
Classificação do medicamento	780 ^†^	16,9	485 ^‡|^	13,0	70 ^‡^	11,6	
Natureza do medicamento	442 ^†^	9,6	234 ^‡^	6,3	38 ^‡^	6,3	
Calibre do dispositivo	796	17,2	753	20,2	128	21,2	
Visibilidade da veia	650 ^†^	14,1	570 ^‡^	15,3	89 ^‡^	14,7	
Palpabilidade da veia	700	15,1	694	18,6	114	18,9	
Preferência do paciente	332	7,2	286	7,7	49	8,1	**0,000** ^||^
Local da cateterização venosa em crianças*							
Mão	550	26,0	510	26,9	83	27,3	
Braço	378	17,8	390	20,1	65	21,4	
Antebraço	514 ^†^	24,3	455	24,0	63 ^‡^	21,0	
Pé	190 ^†^	9,0	139 ^‡^	7,3	14 ^‡^	4,6	
Tornozelo	35 ^†^	1,6	16 ^‡^	0,8	3	0,9	
Cabeça	46 ^†^	2,2	18 ^‡^	0,9	2	0,6	
Pescoço	38 ^†^	1,8	5 ^‡^	0,3	1	0,3	
Não realiza o procedimento em crianças	366	17,3	363	19,1	73	24,0	**0,000** ^§^
Local da cateterização venosa em adultos*							
Mão	631 ^‡^	25,3	685 ^†^	27,7	113	26,9	
Braço	687 ^‡^	27,5	777 ^†^	31,4	140 ^†^	33,3	
Antebraço	941	37,7	916	37,0	154	36,7	
Pé	23	0,9	39	1,6	5	1,2	
Tornozelo	5	0,2	21 ^†^	0,8	1	0,2	
Cabeça	4	0,16	1	0,04	1	0,2	
Pescoço	145 ^†^	5,8	27 ^‡^	1,1	5 ^‡^	1,2	
Não realiza o procedimento em adultos	60 ^†^	2,4	9 ^‡^	0,4	1 ^‡^	0,2	**0,000** ^§^

* Questão que admitiu respostas múltiplas

† Resíduo ajustado > 1,96

‡ Resíduo ajustado < -1,96

§ Teste da razão de verossimilhança

|| Teste Qui-quadrado de Pearson para k amostras independentes

 Os DAVs mais citados foram àqueles com dispositivo de segurança. Com relação ao material destes dispositivos, os enfermeiros mencionaram o poliuretano e o Teflon ^®^ , e no grupo dos técnicos e auxiliares de enfermagem mais da metade não soube informar a constituição dos dispositivos utilizados em suas práticas ( Tabela 4 ). 

 A maioria dos profissionais utiliza o dispositivo de 24 Gauge (G) em crianças e o de 20G em adultos. Avista-se, também, que são adotados mais de um critério para a seleção do dispositivo, sendo os mais listados: calibre do vaso, fragilidade da pele, tempo de uso da terapia e tipo de infusão ( Tabela 4 ). 

 Quanto aos materiais para cobertura e estabilização do DAV, a fita adesiva do tipo Micropore ^®^ foi bastante citada pelos profissionais de Enfermagem, seguida pelo filme transparente. Referindo-se à identificação da CIP, constata-se que menos da metade dos profissionais sinaliza o registro da hora, calibre e nome do profissional responsável pelo procedimento ( Tabela 4 ). 

 Houve diferenças no grupo de enfermeiros e técnicos de enfermagem que utilizavam todos os tipos de materiais do dispositivo, DAV com calibre 22G no público adulto e cobertura transparente. Entre os auxiliares a diferença foi constatada nas categorias Teflon ^®^ e poliuretano, grupo que não sabia o número do dispositivo e que usava IV-Fix ^®^ ( Tabela 4 ). 

**Tabela 3:** - Técnica e procedimentos adotados para inserção de dispositivo vascular periférico pelos profissionais de Enfermagem executantes da cateterização intravenosa periférica (n = 2.584). Brasil, 2022

**Variável**	**Enfermeiro** **(n= 1.214)**		**Técnico de enfermagem (n= 1.166)**		**Auxiliar de enfermagem** **(n= 204)**		**p-valor**
	**n**	**%**	**n**	**%**	**n**	**%**	
**Técnica para garroteamento do membro** *							
Torniquete de uso único	273 ^†^	17,6	355 ^‡^	24,8	68 ^‡^	26,4	
Torniquete universal	675 ^‡^	43,4	594 ^†^	41,6	118	45,9	
Luva ou parte da luva para procedimento	514 ^‡^	33,1	426 ^†^	29,8	69	26,8	
Mãos do profissional	92 ^‡^	5,9	53 ^†^	3,7	2 ^†^	0,8	**0,000** ^§^
**Tempo de garroteamento**							
30 seg	425	35,3	380	33,2	59	29,2	
1 minuto	397	33,0	403	35,2	78	38,6	
1 min 30 seg	69	5,7	91	7,9	11	5,4	
2 minutos	62	5,2	63	5,5	8	4,0	
Não controla o tempo	250	20,8	209	18,2	46	22,8	0,126 ^||^
**Distância torniquete-área de cateterização**							
O mais perto possível do local	582 ^‡^	48,5	502 ^†^	43,9	86	43,7	
O mais distante possível do local	516	43,0	508	44,4	84	42,6	
Tanto faz	103 ^†^	8,6	133 ^‡^	11,6	27	13,7	**0,028** ^||^
**Método de inserção do dispositivo**							
Direto	959 ^‡^	80,5	863 ^†^	76,1	143 ^†^	73,0	
Indireto	200	16,8	218	19,2	38	19,4	
Não sabe	33 ^†^	2,8	53	4,7	15 ^‡^	7,7	**0,002** ^||^
**Máximo de tentativas de cateterização**							
1 tentativa	20 ^†^	1,7	49 ^‡^	4,3	11 ^‡^	5,5	
2 tentativas	597 ^†^	50,0	620 ^‡^	54,2	113	56,8	
3 tentativas	427	35,7	388	33,9	56 ^†^	28,1	
4 tentativas	74 ^‡^	6,2	33 ^†^	2,9	7	3,5	
Quantas forem necessárias	77 ^‡^	6,4	53 ^†^	4,6	12	6,0	**0,000** ^||^

* Questão que admitiu respostas múltiplas

† Resíduo ajustado < -1,96

‡ Resíduo ajustado > 1,96

§ Teste da razão de verossimilhança

|| Teste Qui-quadrado de Pearson para k amostras independentes

**Tabela 4:** - Dispositivos, cobertura/estabilização e registros de enfermagem realizados por profissionais de Enfermagem executantes da cateterização intravenosa periférica (n = 2.584). Brasil, 2022

**Variável**	**Enfermeiro (n= 1.214)**		**Técnico de enfermagem (n= 1.166)**		**Auxiliar de enfermagem (n= 204)**		**p-valor** *
	**N**	**%**	**n**	**%**	**n**	**%**	
**Tipo de dispositivo** ^†^							
Simples	508	34,8	501	35,4	101	40,0	
Com dispositivo de segurança	944	64,7	907	64,0	150	59,2	
Não sabe	7	0,5	8	0,6	2	0,8	0,560
**Material do dispositivo** ^†^							
Teflon ^®^	393 ^‡^	28,4	231 ^§^	20,9	41 ^§^	21,6	
Poliuretano	421 ^‡^	30,5	149 ^§^	13,5	16 ^§^	8,4	
Vialon ^®^	62 ^‡^	4,5	30 ^§^	2,7	7	3,7	
Não sabe	506	36,6	695	62,9	126	66,3	**0,000**
**Calibre do dispositivo em criança** ^†^							
18 Gauge	33	2,0	44	2,9	7	2,8	
20 Gauge	79	4,9	101	6,6	14	5,5	
22 Gauge	440	27,1	390	25,6	58	23,0	
24 Gauge	831	51,1	777	51,1	130	51,6	
Não sabe	242	14,9	209	13,7	43	17,1	0,239
**Calibre do dispositivo em adulto** ^†^							
18 Gauge	316	15,6	314	15,7	50	14,4	
20 Gauge	839	41,3	774	38,6	131	37,9	
22 Gauge	637 ^§^	31,4	708 ^‡^	35,3	111	32,1	
24 Gauge	168	8,3	172	8,6	36	10,4	
Não sabe	70 ^‡^	3,4	37 ^§^	1,8	18 ^‡^	5,2	**0,001**
**Critério para selecionar o dispositivo** ^†^							
Maior disponível	29	0,9	25	0,9	3	0,6	
Menor disponível	64 ^‡^	1,9	38	1,3	2	0,4	
Calibre do vaso	996	30,4	899	31,6	151	31,4	
Fragilidade da pele	704	21,5	617	21,7	108	22,5	
Tempo de uso da terapia	737	22,5	656	23,1	109	22,7	
Tipo de infusão	743	22,7	605	21,3	107	22,3	0,358
**Material utilizado para cobertura/estabilização** ^†^							
Fita adesiva do tipo esparadrapo	318	14,9	298	15,1	52	15,6	
Fita adesiva do tipo Micropore ^®^	616	28,9	600	30,5	119	35,6	
Filme transparente	529 ^‡^	24,8	419 ^§^	21,3	75	22,4	
IV-Fix ^®^	279	13,1	270	13,7	28 ^§^	8,4	
Tegaderm ^®^	387	18,2	382	19,4	60	17,9	**0,026**
**Identificação da cateterização intravenosa periférica** ^†^							
Data do procedimento	94	7,9	88	8,2	19	9,3	
Horário do procedimento	454	38,1	408	37,9	65	31,7	
Calibre do dispositivo	399	33,5	325	30,2	68	33,2	
Profissional responsável	243	20,4	254	23,6	53	25,8	0,214

* Teste Qui-quadrado de Pearson para k amostras independentes

† Questão que admitiu respostas múltiplas

‡ Resíduo ajustado > 1,96

§ Resíduo ajustado < -1,96

## Discussão

Neste estudo, verificou-se variações significativas nas práticas relacionadas à CIP implementadas por enfermeiros, técnicos e auxiliares de enfermagem atuantes nas regiões geográficas do país, algumas em conformidade com o preconizado pelas recomendações vigentes, enquanto outras não condizentes com as melhores práticas para a CIP.

 Foi observado que os enfermeiros são os profissionais que menos orientam os familiares/responsáveis sobre o procedimento de CIP. Neste âmbito, há de se destacar que o planejamento do cuidado em TIV deve envolver a família, pois esta colabora no processo de tratamento e recuperação da saúde de seus entes ^(^ 12 ^)^ . Esta relação deve ser fundamentada em uma parceria que respeita os valores, preferências, sentimentos e necessidades do paciente ^(^ 13 ^)^ . 

 Neste pensar, instrumentalizar o familiar/responsável e/ou paciente sobre a identificação correta desses eventos auxilia a evitar o sofrimento e o desconforto ocasionados por novas tentativas de CIP, que podem resultar em depleção do acesso venoso, medo de agulhas e evasão hospitalar ^(^ 14 ^)^ . 

 No quesito "preparo do paciente pediátrico antes da CIP", de modo geral, os profissionais de Enfermagem deste estudo adotam mais de uma estratégia, além de ser notória a atenção dada ao manejo da dor antes da cateterização. Pesquisa de corte transversal realizada em Unidade de Terapia Intensiva Neonatal de hospital em Fortaleza-CE evidenciou alterações comportamentais e fisiológicas em recém-nascidos submetidos à CIP e que não utilizaram medidas não farmacológicas para alívio da dor ^(^ 15 ^)^ . 

 A cateterização venosa é um procedimento doloroso que traz uma experiência sensorial e emocional desagradável ^(^ 16 ^)^ . Destarte, a equipe de Enfermagem deve torná-la menos estressante, através de estratégias para controle da dor e aplicação de instrumentos de mensuração da dor vivenciada pelo paciente ^(^ 17 ^)^ . 

Na presente pesquisa, os cuidados assépticos antes de cada tentativa de inserção do DAV contrariam os padrões de práticas reconhecidos mundialmente, a exemplo da não higienização das mãos, o não uso de novo material para antissepsia ou de um novo dispositivo. No que tange à troca da luva de procedimento, observa-se uma melhora nos percentuais, mas traz à tona que os profissionais podem estar substituindo a higienização das mãos pelo uso de luvas. No grupo de enfermeiros essa taxa foi menor, mas ainda assim representa um número notável de profissionais.

 A higiene das mãos não pode ser substituída pelo uso da luva de procedimentos. Os profissionais justificam esta troca alegando que há perda do tato no momento da palpação da veia ou da CIP, e muitos utilizam a luva somente quando vão fazer a conexão do DAV com o equipo ou seringa ^(^ 18 ^)^ . 

Diversas pesquisas atestam que a adesão dos profissionais continua baixa mesmo com tantas recomendações sobre a relevância das práticas de higienização antes da inserção do DAV.

 Assim, o olhar sobre a Teoria do Comportamento Planejado (TCP) pode trazer uma luz sobre esta e outras questões relacionadas à CIP. Trata-se de um modelo preditor do comportamento em que três constructos psicológicos (atitude, norma subjetiva e percepção de controle) explicam que a intenção dos profissionais é o antecedente imediato do comportamento ^(^ 19 ^)^ . 

 A atitude liga o interesse pessoal em realizar determinado comportamento aos resultados dele esperados. O profissional de Enfermagem, a partir de suas crenças comportamentais, avalia as consequências da não adoção das práticas corretas de CIP, e o resultado desta análise irá determinar sua intenção ^(^ 19 ^)^ . 

 A norma subjetiva diz respeito a percepção do profissional sobre a pressão social para que o mesmo realize determinado comportamento ^(^ 19 ^)^ . Em muitos contextos laborais, a pressão social do enfermeiro para que a equipe realize as condutas assépticas antes de cada tentativa de CIP é inexistente, sendo que eles próprios não adotam tal postura. 

 Já a percepção de controle demonstra o grau em que o profissional se sente capaz de realizar um comportamento e pode ser influenciada pela atitude e norma subjetiva. Mesmo que o profissional de Enfermagem seja favorável à adoção de práticas adequadas de CIP, um indivíduo pode sucumbir a pressões sociais exercidas pelos demais colegas ou ao baixo controle ^(^ 19 ^)^ . 

 Sobre a avaliação da rede venosa, os profissionais relatam o uso de mais de um critério. O método tradicional e a aplicação de calor foram os mais citados pelos técnicos e auxiliares de enfermagem, enquanto enfermeiros destacam o uso de tecnologias como a ultrassonografia vascular e o Venoscópio ^®^ . 

 No método tradicional, os profissionais são guiados pelos pontos de referência anatômica, inspeção e palpação da veia periférica. Apesar de ser comumente utilizada na prática clínica pelos profissionais de nível médio e técnico, estudo de revisão sistemática constatou superioridade do uso da ultrassonografia em relação à cateterização tradicional ^(^ 20 ^)^ . Este dado ressalta que a tecnologia de visualização da veia permite maior assertividade, agilidade e segurança, menor número de tentativas para o sucesso na cateterização, menos complicações, redução do tempo de duração do procedimento e maior satisfação do paciente ^(^ 21 ^)^ . 

 Um resultado inquietante é que alguns técnicos e auxiliares de enfermagem alegam a utilização da ultrassonografia vascular em suas práticas profissionais, apesar do uso desta tecnologia ser privativo do enfermeiro. Além disso, ainda impera a cultura de que a CIP é um procedimento simples e pode ser executado por qualquer membro da equipe de Enfermagem ^(^ 22 ^)^ . 

 Deste modo, o enfermeiro precisa assumir a responsabilidade legal na inserção do dispositivo periférico, pois associado à complexidade do procedimento há condições que tornam a punção venosa periférica difícil (PVPD), a exemplo de crianças prematuras, desnutridas, obesas ou com doenças crônicas e adultos com alta complexidade assistencial ^(^ 23 ^)^ . Neste último caso, a prevalência de PVPD é de 59,3% e varia conforme o quadro clínico do paciente ^(^ 24 ^)^ . 

A maioria dos profissionais de enfermagem mencionou a utilização de ferramentas clínicas para a avaliação da rede venosa difícil. Apesar do percentual ter sido maior no grupo dos auxiliares de enfermagem, foi observado que estes não sabiam a definição de ferramenta clínica, pois listaram a utilização da anatomia, tecnologia, garrote, dispositivo, dentre outros, como sinônimos.

 A adoção de instrumentos de medida na prática clínica, tais como escalas, protocolos com recomendações, *Bundles* e fluxogramas para avaliar a dificuldade de CIP pode contribuir com a qualidade da assistência de Enfermagem, com consequente redução da taxa de insucesso e da depleção dos vasos sanguíneos, pois os cuidados vasculares são importantes para a preservação da saúde dos vasos ao longo da vida de um indivíduo ^(^ 25 ^)^ . 

Outros cuidados importantes incluem a seleção de dispositivos de calibre apropriado, a escolha do local de veias periféricas que suportem a terapêutica a ser implementada, a técnica de inserção do DAV, e a administração de soluções e fármacos em quantidades e concentrações adequadas.

 Diversos critérios para a seleção de veias periféricas foram apontados pelos profissionais neste estudo. O tempo de uso da TIV periférica e o calibre do dispositivo foram citados por todas as categorias profissionais. Os enfermeiros listaram, ainda, a classificação do medicamento, enquanto os técnicos e auxiliares de enfermagem acrescentaram a palpabilidade da veia. O tipo de solução a ser infundida, o tempo de infusão e as condições das veias também devem ser considerados ^(^ 26 ^)^ durante o tratamento de pacientes hospitalizados. 

Adicionalmente, a equipe de Enfermagem deve monitorar os efeitos e conhecer a natureza dos medicamentos, a fim de evitar complicações locais e/ou sistêmicas. No presente estudo, este critério foi pouco considerado pelos profissionais de Enfermagem.

Outro aspecto relevante pouco citado pela equipe de Enfermagem diz respeito à preferência do paciente. Talvez a comunicação verbal entre profissional e paciente antes da inserção do DAV não seja uma rotina dentro do serviço de saúde.

 No presente estudo, os profissionais de Enfermagem mencionaram as mãos, o braço e o antebraço como os principais locais de escolha para a cateterização venosa em crianças. Estudo descritivo desenvolvido em Unidades de Cuidados Intensivos Neonatais (UCIN) e Pediátricos (UCIP) mostrou que 55,5% dos profissionais de Enfermagem da UCIN e 34,6% da UCIP elegeram as veias do dorso das mãos como primeira opção para CIP ^(^ 27 ^)^ . 

 Estes dados estão em conformidade com a *Infusion Nurses Society* (INS) americana, que recomenda a escolha dos vasos com maior chance de duração de toda a terapia prescrita, como os membros superiores e o dorso das mãos de pacientes pediátricos, evitando-se as áreas de flexão para não limitar a movimentação da criança ^(^ 7 ^)^ . 

 Em contraposição, a INS Brasil traz que a primeira tentativa de CIP deve ser iniciada pela região mais distal, considerando as particularidades de cada fármaco e possíveis complicações ^(^ 28 ^)^ . Indica, também, a escolha de veias da cabeça em crianças com idade inferior a três anos e, caso não caminhem, a utilização das veias do pé ^(^ 7 ^)^ . 

Pesquisa transversal global realizada em 278 hospitais de 47 países com dados de 4.206 crianças identificou a mão (51%; n= 2143) como local mais acessado para a colocação do DAV.

 Entretanto, na América do Norte, Austrália e Nova Zelândia, a fossa antecubital (taxa variando entre 21,4 e 24,5%) foi a região mais apontada ^(^ 29 ^)^ . 

 No atendimento de emergência, essa região é bastante acessada, devido *à* possibilidade de infusão rápida de grandes volumes e, por esta razão, alguns autores recomendam este local por causa do maior calibre das veias ^(^ 12 ^)^ . Em contrapartida, outros não recomendam, pela limitação da mobilidade do membro do paciente, a menos que se utilizem dispositivos venosos flexíveis ou que este seja o único acesso venoso disponível ^(^ 26 ^)^ . 

 Em adultos, as veias cefálica e basílica no antebraço são os locais preferidos para a CIP. Grande parte dos profissionais entrevistados citaram o braço e o antebraço, mas alguns escolheram os membros inferiores como local de cateterização. Este achado, apesar do baixo percentual, causa preocupação, devido ao maior risco de embolia, tromboflebite e infecção decorrentes de CIP em veias da perna/pé de adultos ^(^ 26 ^)^ . 

Depois da seleção da veia, são adotados técnicas e procedimentos para facilitar a inserção do DAV, a exemplo do uso do torniquete, que permite a venodilatação e facilita a visualização e o processo de CIP. A respeito do tempo de permanência do torniquete no paciente, quase a totalidade dos enfermeiros relataram que deixam por 30 segundos, enquanto os técnicos e auxiliares de enfermagem mantêm por até 1 minuto. Não obstante, um percentual expressivo de profissionais não controla o tempo.

 O não controle do tempo pode ocasionar erros diagnósticos como hemólise, elevação do nível de potássio e/ou da dosagem de cálcio, bem como gerar complicações durante a cateterização, como hematomas, formigamento e, em casos extremos, sinal de Trousseau ^(^ 30 ^)^ . 

 Há, ainda, casos de torniquetes esquecidos nas extremidades de pacientes após tentativas de colocação do DAV. Portanto, o enfermeiro deve estar atento a sinais de retenção de torniquete, que incluem dor nas extremidades, formigamento, edema, infusão intravenosa fluindo mal, vazamento no local da inserção do DAV e/ou locais de cateterização ^(^ 31 ^)^ . 

 Outro fator predisponente para a ocorrência de complicações durante a TIV é a técnica de inserção do DAV, pelo método direto ou indireto. Com frequência, os profissionais deste estudo mencionaram o uso do método direto, mas um pequeno percentual de auxiliares e técnicos de enfermagem não sabiam identificar o método de inserção do dispositivo. Evidências sinalizam que o método direto está mais associado à ocorrência de complicações intravenosas ^(^ 32 ^)^ . 

À vista disso, os serviços de saúde têm buscado garantir um cuidado livre de riscos e danos. E neste contexto, os enfermeiros desempenham papel primordial na avaliação do risco de acesso venoso periférico difícil, no intuito de evitar inúmeras tentativas malsucedidas de cateterização.

 Estudo realizado em Portugal evidenciou que enfermeiros necessitam de duas a oito tentativas de cateterização para inserir um DAV com sucesso, com taxa variando entre 19,4% e 23,7% ^(^ 33 ^)^ . Esse número tende a aumentar para uma média de cinco tentativas por pessoa, podendo variar entre uma e 20 tentativas quando se considera todo o período de tratamento do paciente hospitalizado ^(^ 33 ^)^ . 

 A Agência Nacional de Vigilância Sanitária (ANVISA) recomenda até um máximo de duas tentativas por profissional com auxílio de tecnologias de visualização de veias, e limita o número de tentativas a no máximo quatro punções (duas por profissionais diferentes) ^(^ 25 ^)^ . 

 Repetidas tentativas de CIP afetam negativamente a experiência geral do paciente e podem causar danos ao vaso, aumentando a chance de uso de dispositivos de acesso venoso central ^(^ 3 ^)^ . Os profissionais participantes desta pesquisa mencionaram a realização de três tentativas, o que já contraria as normatizações vigentes, sendo esta taxa maior no grupo de enfermeiros. 

 No que concerne às práticas relacionadas aos cuidados com os dispositivos e cobertura/estabilização, observa-se que os materiais do dispositivo mais utilizados pelos enfermeiros foram o poliuretano e Teflon ^®^ . Uma parte dos técnicos e auxiliares de enfermagem usava dispositivo de Teflon ^®^ , e mais da metade não soube informar o tipo de material (um percentual considerável de enfermeiros também se enquadra nesta categoria). 

 O desconhecimento sobre a constituição do DAV aumenta o risco de ocorrência de complicações, visto que as evidências mostram que os dispositivos confeccionados em poliuretano estão associados a baixas incidências de complicações infecciosas quando comparados aos de polivinilcloreto e polietileno ^(^ 34 ^)^ . 

Em geral, os profissionais de Enfermagem utilizam dispositivos de 24G em crianças e 20G em adultos. Nota-se que a escolha do calibre no público pediátrico apresentou um percentual considerável de profissionais que não sabiam a numeração do dispositivo, com destaque para os auxiliares de enfermagem e enfermeiros.

 Para a clientela pediátrica, é recomendável o uso de calibres 22G e 24G, mas um pequeno percentual dos entrevistados utiliza calibres de 18G e 20G. Os dispositivos com menor calibre estão associados a menos complicações, além de causar menos agressão mecânica à parede da veia pela cânula e menor obstrução do fluxo sanguíneo dentro do vaso ^(^ 9 ^)^ , mas essa assertiva deve levar em consideração a idade do paciente e as características da rede venosa. 

As veias da criança são mais frágeis quando comparadas aos adultos, logo, a avaliação dos riscos e benefícios de cada tipo de dispositivo, como calibre, constituição e critérios de seleção, é fundamental. Neste último item, os profissionais de Enfermagem, majoritariamente, elegeram o tempo de uso da terapia como o critério principal para a seleção do dispositivo.

 Após a CIP, os dados salientaram que a maioria dos profissionais de Enfermagem, incluindo o enfermeiro, utiliza a fita adesiva do tipo Micropore ^®^ para estabilizar e cobrir o dispositivo. Este achado é inquietante, visto que a utilização de fitas adesivas não estéreis é uma prática amplamente observada em países em desenvolvimento. 

 É comum observar inúmeros profissionais de Enfermagem que cortam tiras de esparadrapo antes de realizar a CIP e fixam no próprio uniforme, na bandeja ou na mesa de cabeceira do paciente. O propósito da cobertura é proteger o sítio de inserção do DAV e diminuir o risco de infecção, portanto esta prática rotineira contamina a fita após a abertura da embalagem original. A fita adesiva deve ser estéril e trocada sempre que estiver úmida, suja, solta ou com integridade comprometida. Caso haja suspeita de contaminação, a troca deve ser imediata ^(^ 7 ^)^ . 

Outro aspecto significativo após a inserção do DAV é a sua identificação, que permite a comunicação entre a equipe de Enfermagem e possibilita a continuidade da assistência. No presente estudo, nota-se que a equipe não se preocupa com o registro da data do procedimento, pois os percentuais de adoção dessa prática foram muito baixos, inclusive no grupo de enfermeiros. A maioria dos profissionais sinaliza o registro do horário do procedimento, o calibre do dispositivo e nome do profissional responsável no dispositivo.

 A falta de registro da data pode contribuir para a alta prevalência de cateteres ociosos, definidos como não utilizados nas 24 horas anteriores e sem planos para uso nas próximas 24 horas. A literatura aponta que cerca de 14 a 50% dos periféricos são mantidos no paciente “apenas no caso” de serem necessários ^(^ 35 ^)^ . 

 Em algumas instituições, o tempo preconizado para a permanência do DAV é de 96 horas. Para a ANVISA, a troca não deve ser inferior a 96 horas, e a avaliação rotineira da equipe de Enfermagem é que permitirá a decisão em manter o dispositivo por tempo superior, ou quando clinicamente indicado ^(^ 9 ^)^ . 

Ademais, a Organização Mundial de Saúde (OMS) acrescenta que o registro da CIP é um quesito de identificação do paciente e uma meta que garante a segurança nos serviços de saúde e mitiga a ocorrência de erros.

Datar a cateterização permite o acompanhamento do DAV desde a sua inserção, além de possibilitar a conferência da validade nas situações em que a remoção seja programada. E, no caso de alguma complicação, a possibilidade de avaliar os motivos e agir para evitar o agravamento.

Os resultados deste estudo disseminam sobre as melhores práticas relacionadas à inserção do DAV periférico, particularmente no que se refere aos cuidados antes de cada tentativa de inserção, que envolvem o preparo do paciente, avaliação da rede venosa, técnicas e procedimentos adotados, dispositivos, cobertura/estabilização e registros de enfermagem.

Como limitação do estudo, pontua-se a escolha do desenho epidemiológico que analisou as práticas de forma pontual, o que imprime baixo poder em gerar evidências robustas sobre a CIP realizada por profissionais de Enfermagem. Além disso, a amostragem não probabilística interfere na validade externa do estudo.

## Conclusão

A maioria dos profissionais de Enfermagem não envolve o paciente e a família no cuidado, adota estratégias para o preparo do paciente antes da cateterização e não mantém os cuidados assépticos. Na avaliação da rede venosa, o percentual de profissionais que não utilizam métodos de avaliação e/ou ferramentas clínicas foi bem menor.

Muitos profissionais não controlam o tempo adequado de garroteamento e têm dificuldades em identificar a distância correta entre o torniquete e a área de cateterização. Acresce-se que o método de inserção do dispositivo mais adotado é, justamente, aquele que está mais associado à ocorrência de complicações intravenosas, e quase a metade dos enfermeiros e técnicos de enfermagem tentam puncionar o mesmo paciente três ou mais vezes.

 Com relação às características do dispositivo, os profissionais apresentaram conhecimento similar sobre o tipo de dispositivo e calibre usado em crianças e adultos. Há um número elevado de técnicos e auxiliares de enfermagem que não sabe a constituição dos dispositivos utilizados no ambiente laboral. Grande parte dos profissionais utilizava a fita adesiva do tipo Micropore ^®^ para cobertura e estabilização do DAV, e quase a totalidade das três categorias profissionais investigadas não registrava a data da CIP. 

À vista dos resultados encontrados, percebe-se que os técnicos e auxiliares de enfermagem são os profissionais que menos atendem ao que está preconizado nas diretrizes reconhecidas. Entretanto, a prática dos enfermeiros também apresenta desvios em relação às evidências científicas, e sua atuação não foi muito diferente dos profissionais de nível médio/técnico. Assim, foram reveladas fragilidades no cuidado oferecido ao paciente antes da inserção do dispositivo de acesso vascular periférico que podem comprometer a segurança e ocasionar complicações.

Entende-se que é necessária a implementação de ações educativas e de treinamento teórico-prático da equipe de Enfermagem, inclusive dos enfermeiros. Destarte, o conhecimento técnico-científico poderá garantir a eficácia no tratamento e a qualidade do cuidado prestado.
